# Superhydrophobic lab-on-chip measures secretome protonation state and provides a personalized risk assessment of sporadic tumour

**DOI:** 10.1038/s41698-018-0069-7

**Published:** 2018-11-19

**Authors:** N. Malara, F. Gentile, N. Coppedè, M. L. Coluccio, P. Candeloro, G. Perozziello, L. Ferrara, M. Giannetto, M. Careri, A. Castellini, C. Mignogna, I. Presta, C. K. Pirrone, D. Maisano, A. Donato, G. Donato, M. Greco, D. Scumaci, G. Cuda, F. Casale, E. Ferraro, S. Bonacci, V. Trunzo, V. Mollace, V. Onesto, R. Majewska, F. Amato, M. Renne, N. Innaro, G. Sena, R. Sacco, F. Givigliano, C. Voci, G. Volpentesta, G. Guzzi, A. Lavano, E. Scali, U. Bottoni, E. Di Fabrizio

**Affiliations:** 1 Department of Experimental and Clinical Medicine, University Magna Graecia, 88100 Catanzaro, Italy; 2 Department of Electrical Engineering and Information Technology, University Federico II, 80125 Naples, Italy; 3 Institute of Materials for Electronics and Magnetism IMEM CNR Parco Area delle Scienze, 43124 Parma, Italy; 4 Centre of Medical Physics and Biomedical Engineering, Medical University of Vienna, 1090 Vienna, Austria; 5 Department of Chemistry, Life Sciences and Environmental Sustainability, University of Parma, Parco Area delle Scienze 17/A, 43124 Parma, Italy; 6 Department of Health Science, University Magna Graecia, 88100 Catanzaro, Italy; 7 Department of Clinical and Biological Sciences, University of Torino, Torino, Italy; 8 School of Biological Sciences, North-West University, Potchefstroom, 2520 South Africa; 9 Department of Surgical and Medical Science University Magna Graecia, 88100 Catanzaro, Italy; 10 Physical Science & Engineering Division, King Abdullah University of Science and Technology, Thuwal, 23955-6900 Saudi Arabia

## Abstract

Secretome of primary cultures is an accessible source of biological markers compared to more complex and less decipherable mixtures such as serum or plasma. The protonation state (PS) of secretome reflects the metabolism of cells and can be used for cancer early detection. Here, we demonstrate a superhydrophobic organic electrochemical device that measures PS in a drop of secretome derived from liquid biopsies. Using data from the sensor and principal component analysis (PCA), we developed algorithms able to efficiently discriminate tumour patients from non-tumour patients. We then validated the results using mass spectrometry and biochemical analysis of samples. For the 36 patients across three independent cohorts, the method identified tumour patients with high sensitivity and identification as high as 100% (no false positives) with declared subjects at-risk, for sporadic cancer onset, by intermediate values of PS. This assay could impact on cancer risk management, individual’s diagnosis and/or help clarify risk in healthy populations.

## Diagnostics: Proton state of secreted proteins in blood helps identify cancer

A blood test that measures whether molecules secreted by cells contain titratable proton atoms can accurately discriminate between patients who have cancer and those who don’t. Titratable species may in turn influence the protonation state of a solution, i.e. the number of protons added to and the net charge of that solution. A team led by Natalia Malara from University Magna Graecia in Catanzaro, Italy and Enzo Di Fabrizio  from the King Abdullah University of Science and Technology in Thuwal, Saudi Arabia, Francesco Gentile from the University Federico II in Naples, Italy, and Nicola Coppedè from the Institute of Materials for Electronics and Magnetism in Parma, Italy, created an eletrochemical device that can detect faulty metabolism by quantifying the proportion of secreted proteins with and without extra protons—an indicator of abnormal cell division, proliferation and invasion. The researchers tested the device on blood samples from patients with solid tumors and healthy controls. The method identified cancer patients with a high degree of accuracy. If the findings are confirmed in larger trials, the test could help with the screening, diagnosis and management of cancer.

## Introduction

Cancer is the leading cause of deaths worldwide, it entails immense human suffering and very high direct and indirect costs for the health care.^1^ The mechanisms of cancer genesis and development have been heavily investigated. Discovering effective techniques of detection for cancer may improve cancer prevention, transform cancer treatment and may result in longer life expectancy and improved lifestyle for the patient. In medical diagnosis, moderate sensitivity and inadequate precision impair the use of conventional analytical techniques for the assessment of cancer risk. Similarly, medicine functional imaging techniques lack the spatial and temporal resolution necessary to detect a cancer at the early, non-reversible molecular stages of its development. On the other hand, the large variety of cancer types and the different declinations that cancer takes in individual patients are the main cause of the failure of advanced nanomedicine formulations.^2^ Despite recent advances in medical diagnosis, tissue biopsy remains the gold standard for cancer detection and diagnosis. In opposition to classical biopsy, liquid biopsy may respond to the need for a personalized, noninvasive, efficient cancer diagnosis. Liquid biopsy consists in the detection of circulating DNA and/or tumour cells (CTCs) from peripheral blood.^3^ Circulating biomarkers are highly specific but extremely rare and diluted. Devices and assays have been developed for the detection of cancers,^4^ the analysis and monitoring of cancer progression,^5^ for the identification of relevant personalized pharmacological targets.^6^ Nevertheless, no current approach is competitive with clinical diagnosis.

Here, we demonstrate a superhydrophobic, organic electrochemical device that measures the protonation state of secretome derived from liquid biopsy and associates it to cancer formation and progression.The protonation state is a condition resulting in the balance between protonation and deprotonation reactions concerning the set of titratable groups of the protein that are not involved in the structural peptide bonds. The device uses a conductive polymer that is sensitive to the ionic strength of a solution. Sensor surface is patterned at the micro- and nano-scales. Microscale modification of the device enables superhydrophobic effects. Nanoscale modification of the device improves time and spatial resolution of the sensor. The combination of the two spatial scales enhances sensitivity, enables specificity and allows analyte detection at very low concentration levels. The device combines various functions: (i) amplifies differences between species, due to its non-wetting behaviour, through convective Marangoni flows in a superhydrophobic drop; (ii) measures different ion currents associated to certain ionic species in the secretome, through a conductive polymer coating. Thus, specific species can be clustered on the basis of their physical characteristics.

The secretome contains all products of the cell and reflects its metabolism.^7^ The protonation state of the secretome is an indicator of the solution conductivity state of proteins and cell growth,^8–10^ therefore, it can be associated to abnormal cell division, abnormal cell proliferation and invasion and cancer progression.

Analyzing secretome derived from the expansion of non-haematological cells^11,12^ extracted from cohorts of healthy, non-healthy and patients suspected of cancer, the device was able to cluster the different samples and to identify tumour and non-tumour conditions, as well as the transition stage between the two conditions, with high-sensitivity identification accuracy of 100%.

## Results

### The surface-enhanced organic electrochemical transistors (SeOECT)

**Fig. 1:**
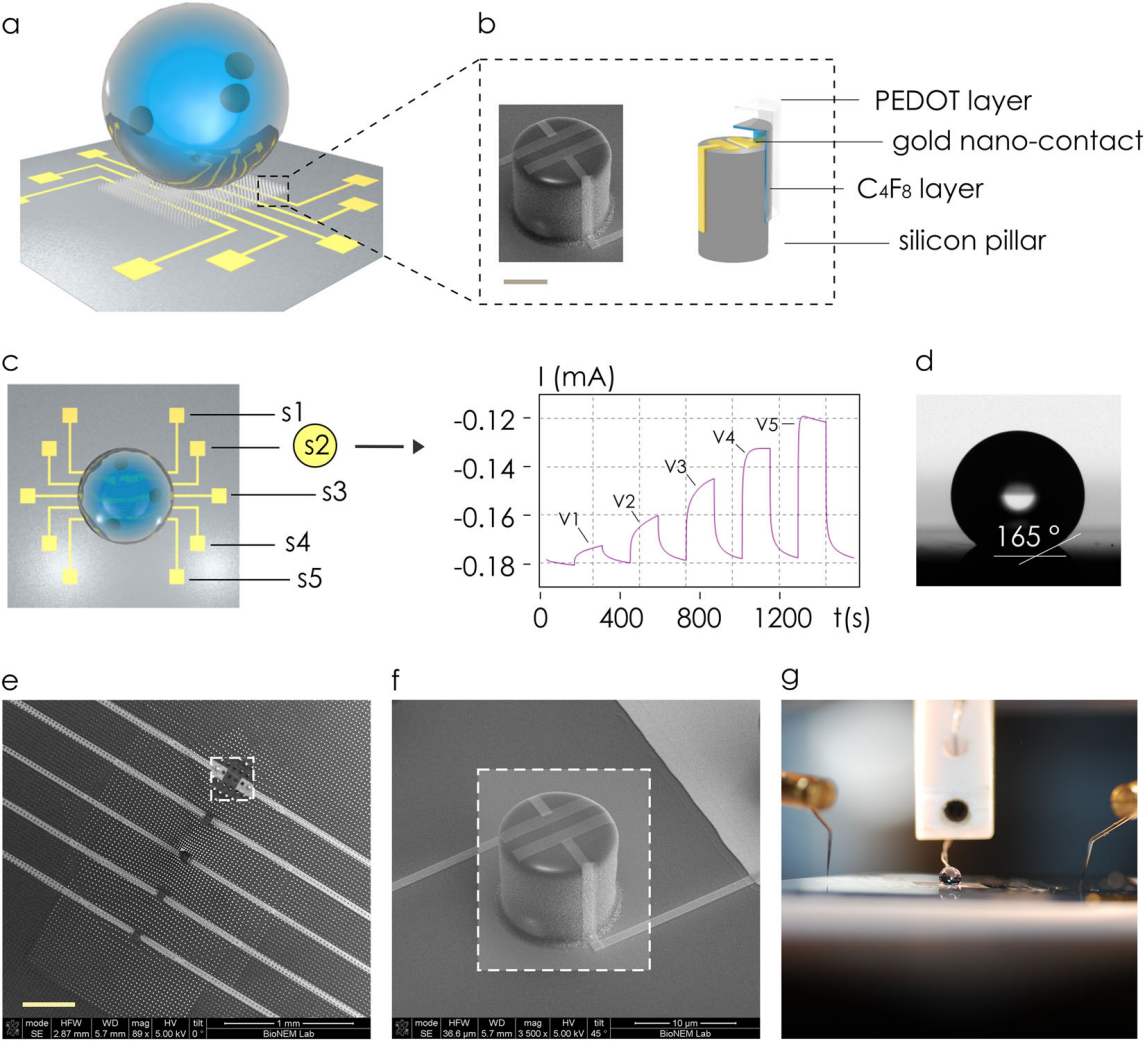
Super-hydrophobic chip incorporates five gold electrodes in a line for site-selective measurements of a solution (**a**). Individual silicon micropillars are modified to incorporate gold nano-electrodes, an intermediate layer of C4F8 and a superficial layer of the conductive PEDOT:PSS polymer, bar in the inset is 5 μm (**b**). Sensors are positioned from the centre (*S*_3_) to the border (*S*_1_, *S*_5_) of the device; the output of each sensor is a function of time *I*(*t*) that reaches a constant value in a characteristic time *τ*; the intensity of *I* is proportional to the externally applied voltage *V* (**c**). Convenient post-processing of a sample enables super-hydrophobicity with contact angles as large as 165° (**d**). SEM micrographs of the device magnified at small magnification factors (**e**). SEM micrograph of the device shows the gold nano-electrodes positioned on the pillar surface for localized sensing (**f**). Due to super-hydrophobicity, biological solutions can be manipulated and connected to an external gate during operation (**g**). The images were partially adapted from https://www.nature.com/articles/srep18992 ^14^

Measurements of the protonation state were performed using a surface-enhanced organic electrochemical transistor (SeOECT) device. The device uses a conductive PEDOT:PSS polymer which is sensitive to the ionic strength of the electrolyte. Nanoscale modification of the device enables the detection of multiple analytes at very low concentration levels. SeOECTs are a third generation of organic thin-film transistors, in which the electrolyte medium is an active part of the device gating. The selective surface of 3D micro and nanostructured device, enhances the properties of an electrochemically active conductive polymer SeOECT. At the microscale (i), the arrays of superhydrophobic micropillars enable manipulation and control of biological fluids (Fig. 1a), whereas at the nanoscale (ii), five pillars, modified to incorporate nano-electrodes (Fig. 1b, c), perform time-resolved and space-resolved analysis of the solution.^13,14^ Each of these electrodes is independently controlled and measures ionic currents (Fig. 1c) that are directly proportional to the charge and to the diffusion coefficient and inversely proportional to the size of the molecules transported in the electrolyte.^15^ Mathematical models can correlate the output of SeOECTs to the physical characteristics of analyzed species in solutions.^15,16^ SEM micrographs of the device show uniformity, reproducibility and verify the fabrication process capability to attain extreme control over shape and size of the micropillars over large areas (Fig. 1e). Pillars are distributed on the substrate to form a non-periodic lattice, in which their relative separation gradually changes from a minimum, at the centre, to a maximum value at the border of the pattern. The non-periodic design induces a nonuniform surface energy density which generates in turn a system of radial forces that recalls the drop to the centre of the lattice^17^ for sample self-positioning. SEM micrographs in Fig. 1f show the system of nano-electrodes on the pillars where nano-gold contacts with sub-micron reciprocal distance generate enhanced and localized electric fields. In the present configuration, the electrodes *S*_1_–*S*_5_ are placed symmetrically with respect to the centre of the device: thus, the electrode/sensor *S*_3_ is at the centre of the chip, electrodes/sensors *S*_1_, *S*_5_ are positioned at the extreme periphery of the pattern or pillars and electrodes/sensors *S*_2_, *S*_4_ lie at an intermediate position between *S*_3_ and *S*_1_, *S*_5_ (Fig. 1c). Convenient post-processing of the chip assures super-hydrophobicity with contact angles larger than 165° (Fig. 1d). An image of the device correctly connected to an external probing station for data acquisition and analysis is reported in Fig. 1g.

### Measuring the protonation state of the secretome by the SeOECT

The secretome is subjected to specific changes in response to fluctuations in physiological states or pathological conditions of individual cells, corresponding to its immediate extracellular volume. In a normal physiological context, the liquid phase of the extracellular volume is characterized by ionic charge content that depends on the PS of the total molecular solute. Perturbations of this ionic charge perturb the PS with changes on electron spin resonance (ESR) signal edited by protonation.^8^ Since the PS depends on the rate of external molecular orbital resonance, consequently, it is strictly linked to the available protonated titratable residues. In 1965, Szent-Gyorgyi drew attention for the first time to the ubiquitous glyoxalase/methylglyoxale enzyme catabolic system that breaks down the 2-oxoaldehyde, a by-product of glycolysis (Supporting figure S1a) and an indirect regulator of both ‘conductivity state of proteins’ and of cell growth.^9,10^ As Supporting figure S1a shows, the cellular metabolism influences the state of intracellular molecular protonation through the balance of glyoxalase/methylglyoxale. The changes in the rate of glycolysis are the first perturbation reflecting changes in cell division or cancer proliferation. The prevalent glucose metabolism in the cancer cells, influencing 2-oxoaldehyde’s catabolism, induces severe modification of their protonation state with respect to normal cells.

**Fig. 2:**
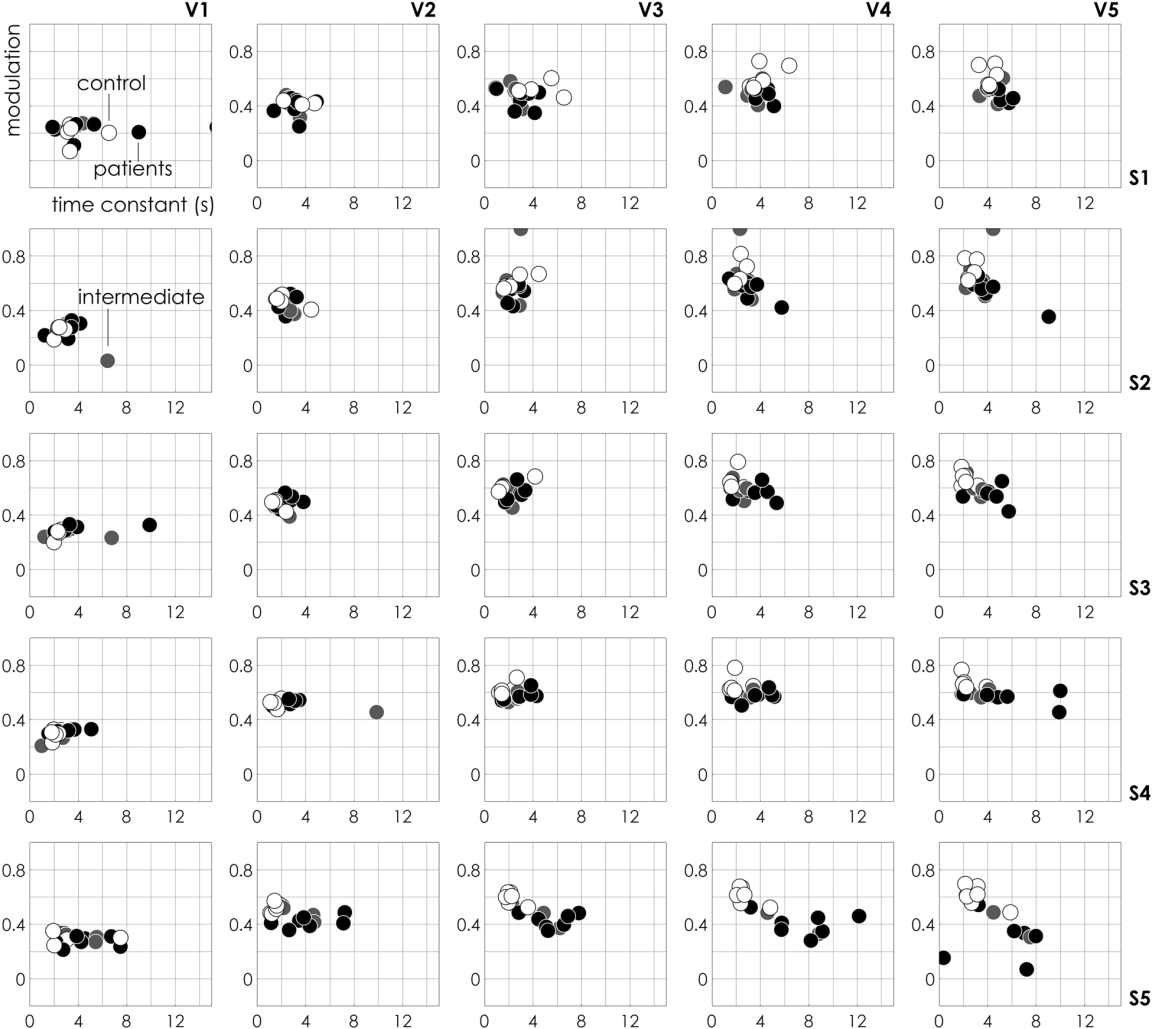
Device response. The response of the sensor, in the analyses of a secretome made by cultures of non-haematological cells isolated by liquid biopsy of control and cancer patients, is described by the sole two parameters modulation *m* and time constant *τ*. *m*–*τ* scatter plots evaluated for different values of applied voltage *V* and sensor position *S* are descriptive of the physical characteristics of the system. Sample separation is operated for high values of voltage and elevated sample numbers. In the diagrams, white label represents control subject (C, without cancer), black label represents patients (P, with cancer) and light-grey label represents intermediate category (I, suspected with cancer)

We used the SeOECT device to analyze the secretome, made by cultures of non-haematological cells isolated by liquid biopsy, of control and cancer patients samples (clinical characteristics were detailed in Data files S1 and S2) in a first cohort of 19 cases (signed with * in Data files S1 and S2). Upon the application of an external voltage at the gate (see Methods), the device measures the ionic activity of charged species in the drop, due to the de-doping effect of positive ions on the conductive polymer. The interaction between positive ions and a conductive polymer results in a current *I*_ds_ proportional to the concentration of ions in the medium. The process is completely reversible, since no irreversible reaction, changing the composition of the solution takes place. The output measurement is a function of time, as reported in Fig. 1c, where the ionic current gradually changes on time, from an initial to a final current value, for a fixed voltage. We define modulation *m*, the difference between the final and the initial values of current, whereas the time constant *τ* of the device, as the time necessary to reach 90% of its final stable signal. In Fig. 2, the modulation for every measured sample is reported as a function of the characteristic time constant for different values of external voltage ranging from *V*_1_ = 0 V to *V*_5_ = 1 V and for different measured points (i.e. electrode positions *S*_1_–*S*_5_) on the chip. Assuming that the behaviour of the SeOECT can be described by only two parameters, i.e. modulation *m* and time constant *τ*, each point of the diagram is the state of the system at a specific voltage, similarly to a graphical representation in the phase space of a dynamical system. In the diagrams, white labels represent control subjects (without cancer), black labels represent patients (with cancer) and light-grey labels represent an intermediate category (suspected with cancer). The control samples are grouped into healthy and non-healthy subjects affected by chronic inflammatory conditions. We observe that, for increasing values of external voltage *V*, control samples gradually shift towards the left-upper region of the plane (large modulation *m* and low time constant *τ*), whereas, on the opposite region, patient samples gradually shift towards the right-lower region of the plane (low modulation *m* and large time constant *τ*). This trend is even more pronounced for electrode *S* positioned at the periphery of the device. For certain combinations of voltage *V* and sensor position *S*, patient and control samples clearly cluster into separate groups. In addition, to confirm the trend, we analyzed a third cohort of nine subjects, four healthy and five affected by chronic inflammatory conditions signed with ** in Data file S1 and reported in Supporting Information 2.

### SeOECT operates sample clustering and classification

**Fig. 3:**
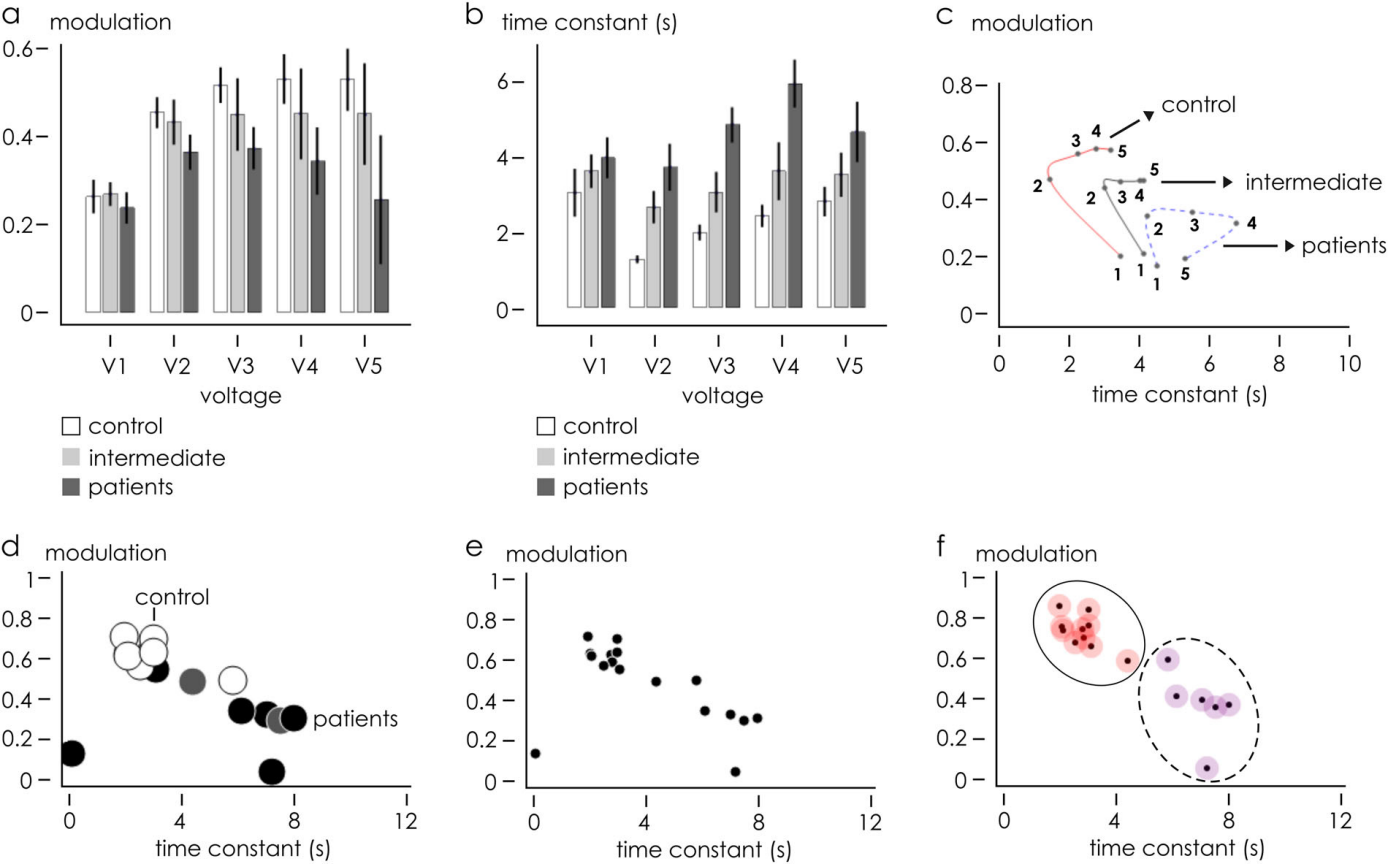
Data analysis of a secretome made by cultures of non-haematological cells isolated by liquid biopsy of control and cancer patients. Bar chart plots of modulation *m* (**b**) and time constant *τ* (**c**) at sensor S_5_ for all samples are reported as a function of *V*. (**d**) Scatter plot of each *m*–*τ* pair is a point whose position varies depending on *V*. On varying *V* in the *V*_1_–*V*_5_ interval, *m*–*τ* doublets delineate a trajectory distinctive of measured samples. Trajectories associated to different sample types, i.e. control, patients and intermediate samples, are not intersecting. *m*–*τ* scatter plot of the whole data set acquired at *V*_5_–*S*_5_ is reported with (**d**) and without (**e**) identification labels. Data set into clusters (**f**), correctly operates assignation of C and P samples into two different groups

Qualitative analysis of Fig. 2 suggests that the device response and sample separation is optimized for sensors positioned at the periphery of the device where Marangoni convective flows are maximized, in agreement with previously reported studies,^14,18^ and for high values of voltage Vg, i.e. an externally applied driving force. We focused the analysis on sensor S_5_. The bar chart in Fig. 3 reports, for different values of voltage Vg = 0.2–1 V, the modulation (b) and time constant (c) values of control and patient samples, extracted from the scatter plot reported in Fig. 2. It is noticeable that, with the exception of the first coordinate *V*_1_ = 0.2, (i) modulation is higher and (ii) time constant is lower in control samples, compared to patient samples. In control samples, *m* varies from 0.521 for Vg = 0.2 V to 0.606 for Vg = 1 V. Conversely, in patient samples, *m* varies from 0.418 to 0.293 over the same voltage range. As for time constant *τ*, in control samples and in the same voltage range, the system moves from *τ* = 1.460 s to *τ* = 3.215 s and then reaches the steady state, in comparison with patient samples where the signal reaches its final value *τ* = 4.257 s for Vg = 0.2 V and *τ* = 5.329 s for Vg = 1 V. These differences are statistically significant for all considered voltages (*p* *<* 0.05, Fig. 3a, b). Since high *m* and low *τ* are indicative of fast-response systems, these data suggest that the products released by control cells in the secretome were more protonated than the products released by cancer cells. A condition of high PS (secretome) generated a final solution characterized by a higher ionic density or a more electrically active secretome, with respect to patient cells. A lower PS and a lower conductivity characterized the species secreted by cancer cells.

Figure 3d represents the scatter plot in *m–τ* diagram for samples with Vg = 1 V and electrode number *S*_5_, where differences between groups are maximized. The plot shows as the samples are correctly classified into groups, on the basis of a priori knowledge of their original populations belonging. Differently, unlabelled points are presented in Fig. 3e, where nonetheless the set of measurements falls into well-defined clusters. A density-based, unsupervised clustering^19^ of unlabelled data presented in Fig. 3e was used to classify elements into categories on the basis of their similarity. The algorithm, determined cluster centres on the areas characterized by a density of points higher than their neighbours and by a relatively large distance from points with higher densities. Points were then coloured according to the cluster of the assigned group (Fig. 3f). The algorithm identified two principal clusters. Upon cluster classification, we found that, in the initial control set of patient points, only one control and one patient element were assigned to incorrect clusters with a classification error *e* = 1/14 ~ 7%. The very low mismatch between the original data sets and their unbiased classification into clusters using the described clustering algorithm confirms that the SeOECT device operates sample identification and tumour detection with high accuracy, high sensitivity and specificity. Nevertheless, device specificity can be improved even further using the device response in combination with a convenient statistical and PCA analysis of data.

### PCA-guided analysis of data

The device measures ionic current transients at five different positions on the substrate and five different values of voltage. The output of the device is lumped into two variables, modulation *m* and time constant *τ*. Even without considering external environmental conditions, the number of potential combinatorial variations through which one can represent the response of the system is 75!/(73! 2!) = 5550. It is nearly impossible to test all combinations of voltage V, sensor position S and modulation and time constant labels, to screen them for the best and more informative representation of the response of the device. While this device offers a wide investigation for improved diagnosis of cancer, at the same time, it poses the problem of selecting the most representative coordinates. In this context, statistical analysis and principal component analysis of data can be used as a preliminary reference for finding representative patterns in the data and to optimize the sensor response.

**Fig. 4:**
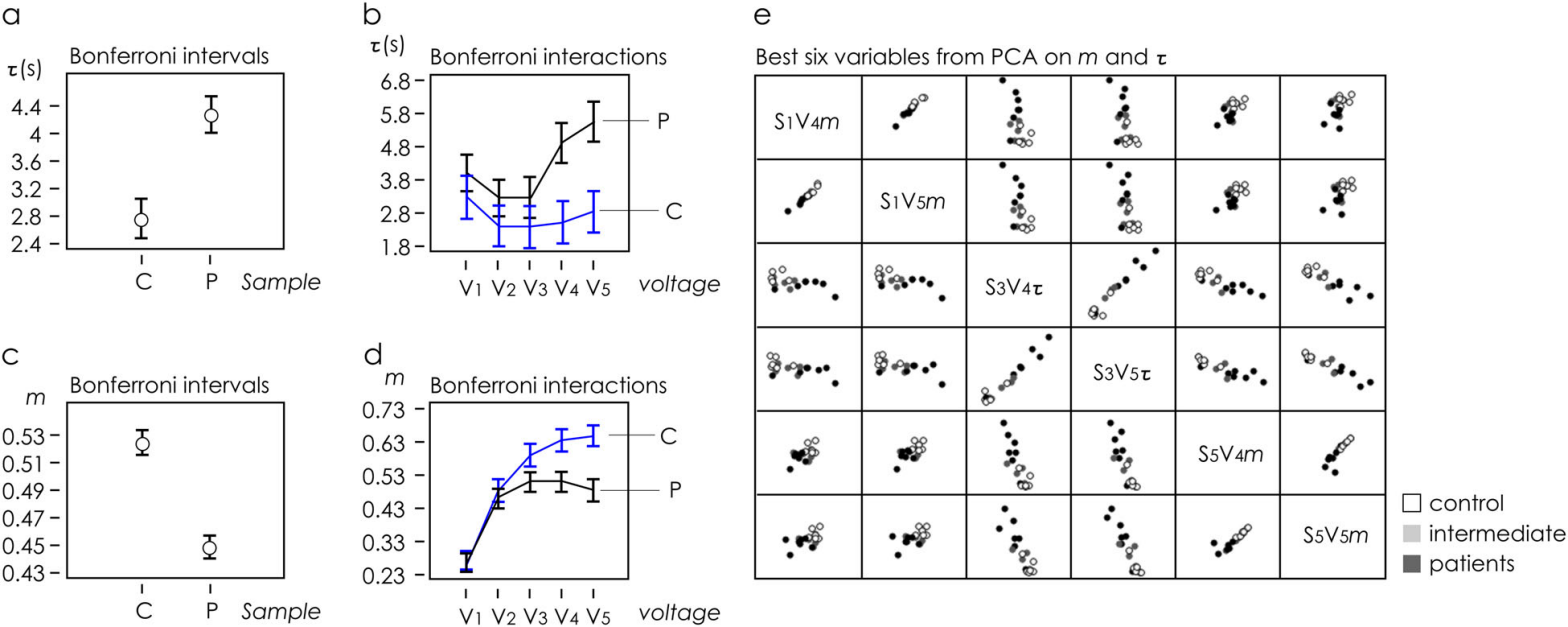
Data set of the secretome made by cultures of non-haematological cells isolated by liquid biopsy of control and cancer patients measured through the device was subjected to ANOVA analysis. Graphical ANOVA from Bonferroni post hoc test shows control C dissimilar from patients P samples with a 95% confidence interval and 5% significance level, for both time constant (**a**, **b**) modulation (**c**, **d**) measured variables. Interaction plots carried out on time constant (**b**) modulation (**d**) outputs, showing significant discrimination between C and P samples for values of potential at the gate *V* higher than *V*_3_. PCA analysis performed to reduce noise and extract from the whole data set combinations of voltage and sensor number, *V*–*S*, in correspondence of which sample separation may be optimized (**e**)

**Fig. 5:**
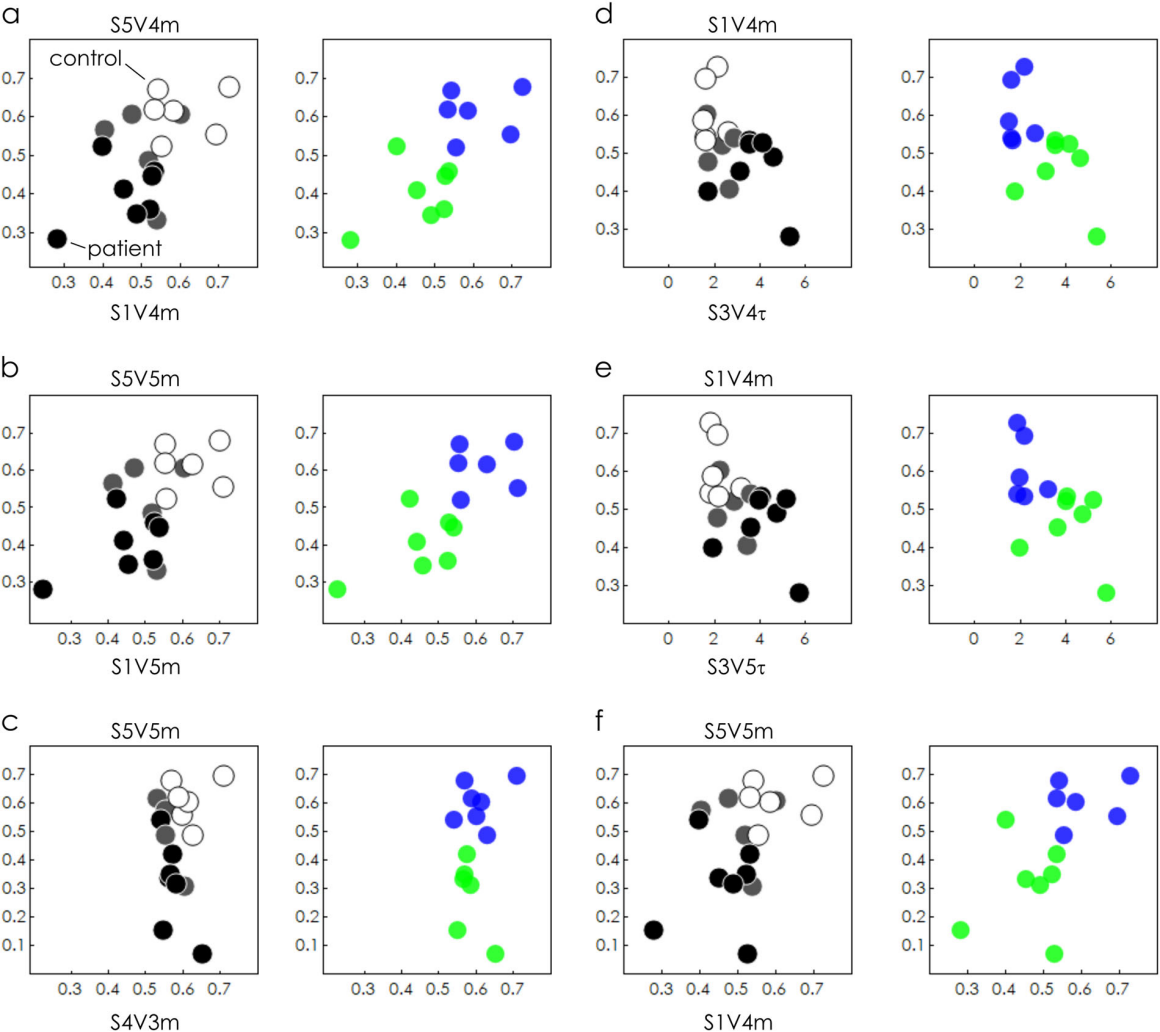
Data clustering and sample identification. On the basis of PCA analysis, we selected the best combinations of voltage, sensor position, modulation and time constant that optimize the response of the device and maximize separation of the sample of secretome made by cultures of non-haematological cells isolated by liquid biopsy of control and cancer patients, between variables (**a**–**f**). For all considered configurations, data were clustered into control and patient groups with a specificity up to 100% with the exception of configuration (**c**), where matching the efficiency of classification is nearly 93%

The whole data set was subjected to Multifactorial ANalysis Of VAriance (ANOVA) in order to individuate the experimental variables significantly correlated to the sample, i.e. control (C) or patient (P) cells, using the time constant *τ* and modulation *m* outputs from the five sensors (Supporting Information 3). The classification factors for ANOVA were the applied potential at the gate Vg and sample typology. Multifactor ANOVA was carried out on *τ* outputs, evidencing a significant effect of both Vg and sample variations. Graphical ANOVA from Bonferroni post hoc test is reported in Fig. 4a. Figure 4b shows the interaction plot from the ANOVA carried out on the *τ* outputs, evidencing a significant discrimination between C and P samples for Vg values higher than *V*_4_. Analogous multifactor ANOVA carried out on modulation outputs *m* also evidenced a significant effect of both Vg and sample variables. Graphical ANOVA from Bonferroni post hoc test is reported in Fig. 4c. Figure 4d shows the interaction plot from the ANOVA carried out on the modulation outputs, evidencing significant discrimination between control and patient samples observed for Vg values higher than *V*_3_. On the basis of these findings, principal component analysis (Supporting Information 3) was carried out for both modulation *m* and time constant *τ*, including independent outputs from five sensors and excluding from the data set the measurements performed at non-significant values to reduce noise in the data-modelling procedure. Selection of best PCA outputs led to the identification of optimal combinations of electrode position and applied voltage (Fig. 4e) that were not otherwise immediately identifiable through direct visualization of data. In Fig. 5a–f, we report scatter plots of data within six different frames of references (*S*_1_*V*_4_ *m*–*S*_5_*V*_4_*m*), (*S*_1_*V*_5_*m*–S_5_V_5_*m*), (*S*_4_*V*_3_*m*–*S*_5_*V*_5_m), (*S*_3_*V*_4_*τ*–*S*_1_*V*_4_*m*), (*S*_3_*V*_5_*τ*–*S*_1_*V*_4_*m*) and (*S*_1_*V*_4_*m*–*S*_5_*V*_5_*m*) extracted from the best PCA variables. At the side of each scatter plot, we report unsupervised clustering of sample data performed using the algorithms described in the precedent section. For the considered configurations, all data points are correctly categorized into *patient* and *control* groups with 0% classification error and 100% identification, with the exception of the configuration (*S*_4_*V*_3_*m*–*S*_5_*V*_5_*m*), where the matching between the experiments and the prediction of the model has an efficiency of nearly 93% (the clustering algorithm used was detailed in Supporting Information 4). Similar results were obtained on analyzing a third independent cohort of eight patients (signed with *** in Data files S1 and S2), as described in a separate Supporting Information 5.

### Biochemical characterization of secretome and clinical transfer

To obtain a complete understanding of the composition of molecular products released by cultivated cells into extracellular space, we collected the medium conditioned for 2 weeks. In particular, we used a protocol involved in the isolation of circulating non-haematological cells from peripheral blood in cancer patients previously reported,^11,12^ thus identifying a specific density phase for CTCs in peripheral blood. The cells isolated from this phase are then put to seeding, as previously described^11,12,20^in a specific culture medium and expanded for 14 days. The short-time cultivation protocol was optimized during many experiments, which allowed us to pinpoint the time required to show up the proliferative capacity of the transformed cells and to preserve their original phenotypes. In order to save the heterogeneous composition of the CTCs, our approach does not make any selection of phenotypes during the initial phase. In fact, we expect the circulating tumour progeny to be made of cancer cells with an epithelial or mesenchymal or hybrid epithelial–mesenchymal phenotypes. The same protocol was applied in healthy and non-healthy patients to isolate circulating non-haematological cells. The non-haematological cells expanded in vitro were characterized for the expression of specific cancer markers, as reported in Supplementary Information (Supporting Figure S1b–g).

**Fig. 6:**
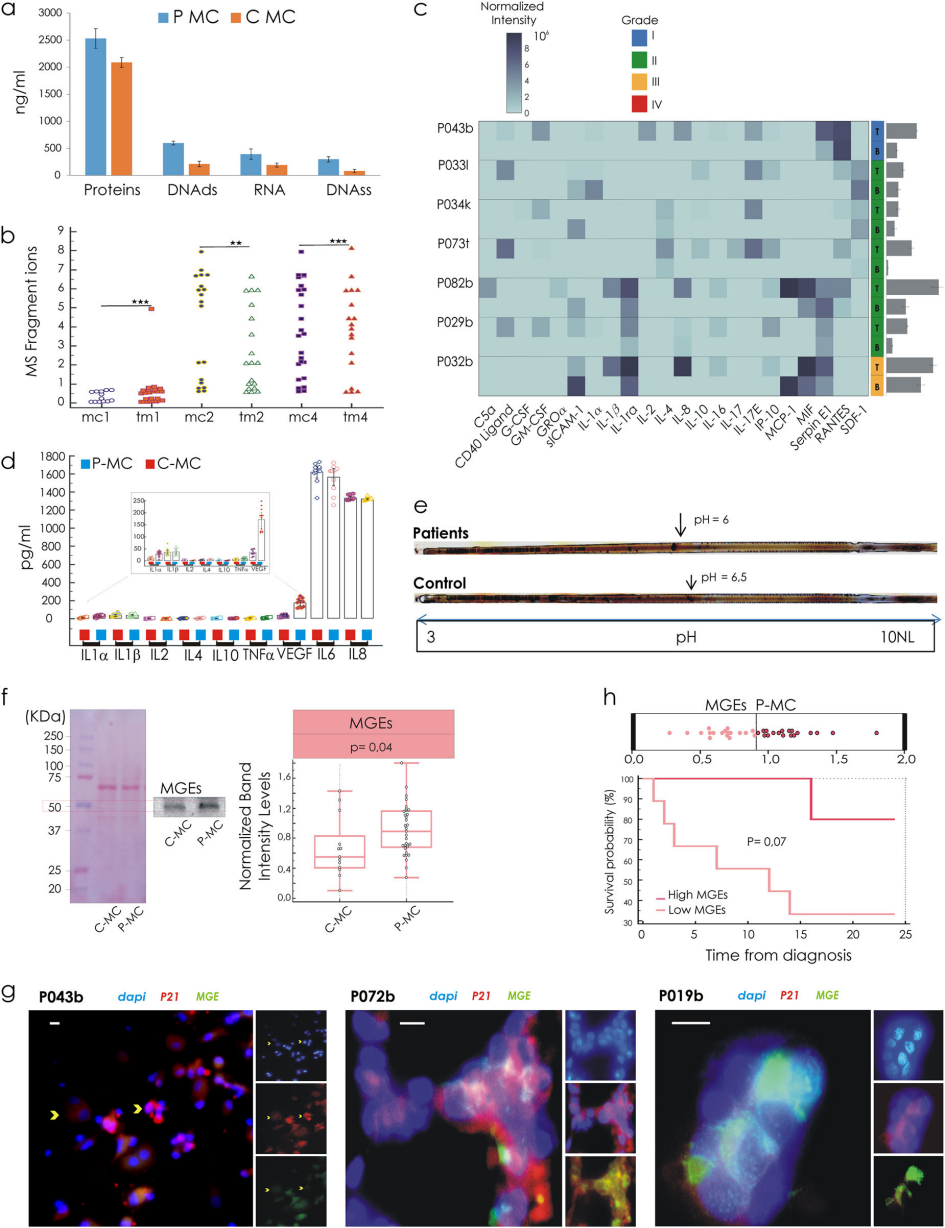
Characterization of secretome in conditioned medium by blood-derived cultures 14 days aged. **a** Spectrophotometric quantification in the secretome of proteins, DNA double strands, RNA and DNA single strand. **b** Correlation between in vitro (conditioned medium (MC)), and in vivo tumour microenvironment (interstitial fluid phase of tumours (TM)) in the same patient, **r = 0.8; ***r = 0.9. **c** of the MS fragment ions by mass spectrometry. **c** Heatmap shows cytokines in a conditioned medium: row represents a patient and column a cytokine. Color intensity represents the levels of cytokine. Each cytokine in the data matrix is obtained through averaging two values quantified on cytokine-array membranes. The error bars are the sum of standard deviations between two values of pixel intensity for each of the patients. **d** Plots by repeated measures ANOVA show distributional levels of the cytokines in control and patients’ groups (*p* < 0.05). **e** Isoelectrofocusing assay on conditioned medium shows the pH value at which the net surface charge switches its sign. **f** Comparative levels of methylglyoxal (MG) glycation end products (MEGs) in MC assessed through immunoblotting. **g** Intracellular localization by immunofluorescence with antibodies against MGEs (green) and p21 (red) in breast cancer cases (reported in Data file S2). Scale bars 10 μm. **h** Kaplan–Meier curves show a worst prognosis for patients with secretome content high levels of MGEs

The principal constituents of BDC’ secretome (BDCS) were proteins, 2.53 ± 0.18 mg/ml and 2.09 ± 0.91 mg/ml; DNA double strands, 599 ± 34 ng/ml and 211 ± 50 ng/ml; RNA, 393 ± 95 ng/ml and 191 ± 36 ng/ml; DNA single strand, 298 ± 48 ng/ml and 81 ± 31 ng/ml measured, respectively, in patients and the control group (Fig. 6a). The BDCS from patients was richer in any component compared to control BDCS (p = 0.001) and its protein content correlated with the grade (*r* = 0.6). We were intrigued by a possible qualitative correlation between BDCS and the interstitial fluid phase of tumours (TM). TM is a component of the internal milieu of a solid tumour and the present proteomic technologies validated it as a valuable source for specific tumour marker candidates.^21^ TM was isolated directly from the fresh tumour specimens, as described in Raso et al.^22^ A strong correlation (*r* = 0.9) between medium conditioned (MC) and TM in the same patient (Fig. 6b) was found applying mass spectrometry (MS) carried out under positive and negative ionization mode. In addition, we compared MC and TM for pro-inflammatory cytokine levels, including interleukin-1 (IL-1a, IL-1b and IL-1ra), IL-2, IL-4, IL-8, IL-10, IL-16, IL-17, IL-17E, C5a, G-CSF, GM-CSF, MCP-1, MIF, ICAM-1, GROα, IP10, SERPIN E1, RANTES and SDF-1 (Fig. 6c). The correlation found for each patient ranged from *r* = 0.4 to *r* = 0.7. We therefore compared the control group with the patient group in terms of the total production of cytokines, evidencing a significant difference between the two groups (*p* ≤ 0.005) (Fig. 6d).

To confirm the difference on the device data in terms of secretome protonation state (SPs), within MCs collected from the two analyzed groups, an isoelectrofocusing assay was performed. It was observed on different values of an isoelectric point between the two clinical groups, as shown in Fig. 6e. Based on our first hypothesis, we explored the relationship between metabolism and SPs through the quantification of secretome glycation end products. An increased level of methylglyoxal (MG) glycation end products (MEGs), prevalently derived from the MG reaction with proteins, was found in the patients’ secretome (Fig. 6f) (p < 0.05). Expanded cells were analyzed in terms of cell cycle phase distribution focusing on the S-phase referencing to their proliferation rate (Supporting Information 1). The patients with S phase > 50% were characterized by high levels of MEGs (*p* = 0.002). Immunofluorescence assay (Fig. 6h), using antibodies against MGEs and p21 (CIP1) showed that the prevalence of expanded CTCs with nuclear expression for p21 expression did not display cytoplasmic accumulation of MGEs. These features were not supported by a significant correlation with S-phase value. Finally, Kaplan–Meier curves and an assessment of survival data were performed. The patient with a secretome with high MGE levels had a worse prognosis (Fig. 6h); therefore, no significant difference in terms of disease-free survival was found.

### Difference in antioxidant defence profile of the samples separated by the SeOECT

**Fig. 7:**
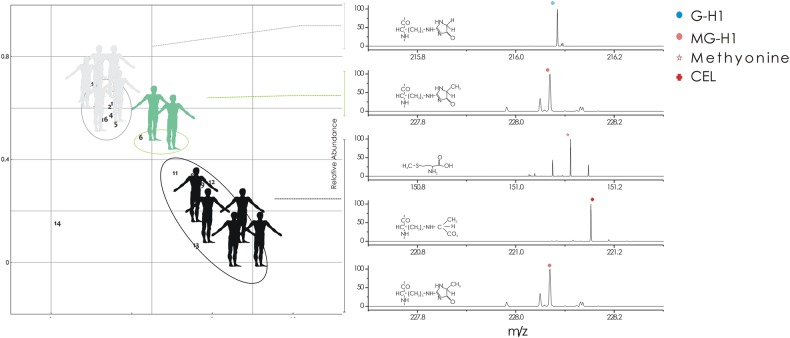
Schematic drawing of samples’ trace and correspondent MS profiles. The trajectory moves from control (grey) to patients (black). Intermediated samples (green) are localized at approximately the halfway point. The right-hand image shows mass spectrometry results performed on MC samples from control and patients groups. High-resolution single-ion monitoring MS, shows the pseudo molecular ion signal of G-H1, MG-H1, methionine and CEL, corresponding to, respectively, ([M+H]+), ([M−H]−), ([M+H]+) and ([M+H]+)

To better establish the role of MGEs in the cancer pathogenesis, we investigated the early-stage protein glycation and advanced-stage protein glycation biomarkers, the nitrosation and oxidative stress^23^ (Supporting Table S1) in MCs separated by SH-BioChip. The early-stage protein glycation products^23^ are due to direct interaction between glucose and the amino groups of lysine residue side chains, as well as N-terminal amino acid residues, leading to a Schiff’s base adducts, commonly defined as fructosyl-lisine residues (FL). These early-stage glycation adducts are relatively rapidly reversed. The advanced-stage protein glycation products are stable end-stage adducts formed by the interaction between endogenous a-oxoaldehyde metabolites, methylglyoxal and a minor contributor glyoxal with different site-specific distributions on proteins (Supporting Figure S1a).^23^ A major advanced-stage glycation product found in human is the methylglyoxal-derived hydroimidazolone, N-(5-hydro-5-methyl-4-imidazolon-2-yl)-ornithine (MG-H1)^24^. A further minor methylglyoxal-derived product is the stable N-(1-carboxyethyl)lysine (CEL) which is mainly formed in the non-enzymatic reaction between methylglyoxal and lysine. Glyoxal advanced-stage glycation products are represented by (N2)-G-H1 (G-H1), N-(1-carboxymethyl) lysine (CML) and (H8)-GOLD (GOLD). The markers of oxidative stress MetSO and dityrosine with the marker of nitrosative stress, 3-nitrotyrosine, were determined^24^ by triple-quadrupole MS. In the control group, clustered in the upper-left region of the chip platform, the correspondent MCs assayed by MS were prevalently (75%) characterized by total absence of early and advanced-stage protein glycation products and nitrosation and oxidative stress biomarkers. Moreover, 20% of samples were characterized by a unique type of advanced-stage protein glycation glyoxal derived (GH1) (Fig. 7). In the case of MCs from patients group, clustered at the bottom-right part of the SH-BioChip plane, 60% of samples were MG-H1 and CEL positive where 20% presented also FL, whereas the remaining 40% was positive for G-H1 and GOLD. Only the group of patient samples displayed a compromised antioxidant defence evidenced by relative peaks for dityrosine and 3-nitrotyrosine.

### SeOECT reveals personalized risk of sporadic cancer

Two samples from the control group showed an intermediate behaviour on the chip. Within the range of the considered voltage length, intermediate samples revealed modulation and time constant values lying approximately halfway between control and patients samples (Fig. 3a–b). Intermediate samples were statistically different from both, the control and patients’ samples, with *p* < 0.10 for modulation and *p* < 0.05 for time constant. A curve or a trajectory that describes the evolution of the system as a function of voltage can be obtained by reporting the centre of mass of samples in the same *τ–m* diagram (Fig. 3c) for different values of voltage. This graphical representation of the system may unmask the otherwise hidden data patterns and highlight differences between different data sets. For the present configuration, similar non-overlapping diverse trajectories confirmed that the analyzed samples originated from different populations representing specific clinical cases. In a more sophisticated evolution of the technique, trajectories in a phase-space diagram obtained by using the SeOECT may reveal variations in the normal course of the physiological/pathological performance of the subjects. The peculiar behaviour of the intermediate samples subgroup suggested an intermediate value of PS with respect to the two major clinical groups analyzed. Intermediate subjects could be identified as 'subclinical entities' in which protein biomarker profile characterized by peaks for MGH-1, CEL and MetSO oxidative stress biomarker (Fig. 7), as well as the position on the SeOECT, revealed a profile for reactive dicarbonyl detoxification and antioxidant defence, under siege, but not collapsed. To date, only one of these two subjects, after 80 months, was diagnosed with melanoma in situ (Clarke level 1).

## Discussion

The device used in this study is an electrochemical transistor, which allows for direct current measurement that is proportional to the ionic strength of the solution. Micro and nanoscale device architecture introduces additional degrees of freedom in the system, enabling sample manipulation, through Marangoni convective flows, and time- and space-resolved sample analysis at the nanoscale. The intensity of Marangoni flows in a point of the device depends on (i) the curvature of the drop, (ii) the distance of that point from the centre of the drop and (iii) the gradient of temperature from the substrate to the solution in the drop. However, it is to be noted that (i) the curvature of a drop is the inverse of its radius, φ = 1/*R*; thus the smaller the drop, the higher the Marangoni effects. Nevertheless, drops with a radius smaller than ~1 mm (that corresponds to a volume *V* ~ 4 μl) cannot be easily spotted on the device and can easily be manipulated or controlled. Moreover, they may contain few target molecules that hamper the readout of the device. Thus, the curvature of a drop may be limited by the implementation of the device and at the—current state of the art—the use of exceedingly small drops (i.e. nano-drops), can be impractical.

(ii) If the curvature (radius) of the drop is fixed, the maximum distance at which a sensor can be positioned is determined by the angle of contact ϑ of the drop with the superhydrophobic device. The radius *r* of the circumference at the solid–liquid interface is *r* = *R* (4/ξ)^1/3^ sinϑ, with ξ = (1 − cosϑ)^2^ (2 + cosϑ), that yields *r* ~300 μm for ϑ = 160° and *R* = 1 mm. Thus, ~300 μm is the maximum distance between the centre and the border of the device in standard operation conditions. Notice that *r* is limited by *R*. If one wants to maintain *R* low, in order to increase the curvature of the drop, then *r* will be necessarily small, with limited leverage of Marangoni flows. Consequently, acting on the geometrical constraints of the device does not appear to be a promising strategy for improving efficiency.

(iii) The intensity of thermo-capillary-driven Marangoni flow in a small droplet depends on the difference between the temperature inside the drop and the temperature on the substrate −∆*T* = *T* − *T*s. While small ∆*T* normally results from the differences of heat conductance between the solid substrate and the liquid phase in the drop, additional ∆*T* can increase even further the intensity of Marangoni flow, maximizing the response of the device. Additional ∆*T* can be obtained by placing the device on a hot plate with accurate control of the temperature at the surface of the plate.

Thus, while Marangoni flows can be in theory maximized using sensors positioned far away from the centre of the device in small drops, in practice, the most effective strategy for increasing the response of the device is to use controlled temperature gradients at the device–solution interface.

On the other hand, the convenience of using multiple sensors in the device is that

- They increase the statistical significance of the analysis;
- They generate large data sets that can be analyzed using PCA or other statistical analysis techniques for finding the best combinations of variables that minimize uncertainty and maximize the response of the device;
- They enable to resolve complex mixtures where the number of unknown species is equal to the number of sensors.

The combination of different length scales allows signal amplification, increases resolution and improves sensitivity of the device. The device is thus capable of registering otherwise undetectable changes in the protonation state of the secretome that, in turn, are clues of cancer genesis.

The surface-enhanced organic electrochemical transistor (SeOECT) allowed the identification of samples based on the protonation state of their secretome.

The combination of two variables, *m* and *τ*, reduces uncertainty and the likelihood of incorrect assignments, thus improving analytical reliability. The different trajectories that in a phase-space diagram can be clearly associated to healthy, non-healthy and precancerous samples, highlight the relevance of the protonation state of the secretome in evaluating the cancer state.

The results were confirmed by independent phenotypic and biochemical analysis of 60 non-haematological cell cultures. Metrics measuring clinical differences between samples were used to resolve groups and patient cases and assigned to correct clusters with small classification errors. This is the result of an initial selection of patients and control subjects that excluded from the analysis possible interference cases (i.e. patients with constitutional dysmetabolic glucose pathways). Thus, accurate preselection of patients is relevant for the correct matching between the prediction of a model with true clinical and biological data.

Taken together, the results support the idea that the protonation state of the secretome, which refers to the microenvironment of circulating non-haematological cells, reflects intracellular changes in the levels of methylglyoxal glycation end products, which are relevant actors of the glycolitic pathway. This concept is supported by findings on the characteristics of the secretome made from expanded non-haematological cells isolated from the liquid biopsy of control subjects and patients. To clear how those secretomes are more or less 'electrically active', we used two parameters, i.e. *m* and *τ*. Since high *m* and small *τ* are indicative of fast-response systems, these data suggest that the products released by control cells in the secretome were protonated more than the products released by cancer cells. Conditions of high PS of the solutes dispersed in the secretome generate a final solution characterized by a higher ionic density or, in other words, higher electrical activity and conductivity that are relieved by the 'controlled perturbation' performed by the SeOECHT. The SeOECHT highlighted a lower PS and a lower conductivity characterizing the protein species secreted by cancer cells. These data suggest that the secretome made from expanded non-haematological cells isolated from the liquid biopsy of healthy subjects presents a faster kinetic diffusion than the secretome of patient cells. These physical conditions are even a biological explanation of the cancer process. The metabolic switch occurring within the cancer cells consists in an increase of the cytosolic glycolytic pathway that avoided the mitochondrial stations. This behaviour is responsible for a cytoplasmic increase of reactive aldehyde’s concentration. These reactive molecules are able to interact with titratable amino acids exposed by the building block of all proteins. The set of titratable groups of the protein is not involved in the structural peptide bond, thus being capable of protonation and deprotonation. The amount of charged titratable group of the protein affects the electrostatic properties of the solution in which they are contained. The higher concentration of the aldehydes (i.e. methylglyoxal, glyoxal) within the cytoplasmic cancer cell favours the interaction with titratable side chains to build final molecular complexes, i.e. the adducts. The adducts have a density of electric charge higher in the internal part of the molecule with respect to the surface and are characterized by lower conductivity and lower electrical state rather than the native protein, with an influence on the electrostatic properties of the cytoplasm, and of the secretome, when released in the next microenvironment.

In addition, the results showed in Fig. 6 demonstrate that the secretome of circulating cancer cells could be considered a good reference of the primary tumour interstitial fluid, further confirmed by the strong correlation between the levels of methylglyoxal and the data survival of cancer patients. In Fig. 7, we highlighted the link between the metabolic switch occurred in the non-haematological cells and the impact of this on the oxidative defence, showing a 'gradient' in the levels of the glycation products comparing the healthy with cancer patients, with an inverse behaviour for the levels of the reduced forms of the antioxidant molecules.

In cancer patients, the presence of circulating tumour cells in the liquid biopsy and relative culture, conditioned the presence of high levels of glycation products. The circulating cancer cells represent the most specific biomarkers actually available and detectable by liquid biopsy. The high specificity of this biomarker is compromised by the low sensitivity of the current methodology used to detect them. Cell-search, Veridex and other methods using an immune-selective approach underestimate the CTC quantification irrespective of the heterogeneity of this kind of population. The present device is able to discriminate cancer and healthy patients as a function of the presence of the CTCs revealed through the physical characteristics of their metabolic product released in the culture medium. The metabolic products became here the reference for the presence of circulating cancer cells in the correspondent culture. This approach overcomes the problematic relative to the heterogeneity and the loss of antigen on the cell surface that are in part responsible for the failure of the methodology followed in the immune selection of these cancer cells. The biosensor detects and characterizes the metabolic species produced by specific biomarkers represented here by the CTC and its secretome.

The case of intermediate values of PS measured with the device deserves to be discussed even further. Samples with intermediate values of PS identify a fuzzy phase in which the process of cell transformation is started at a metabolic level but is not evident in order to the classical indicators of cancer (cell atypia, genetic alterations and clinical evidence). Thus, intermediate PS values highlight an intra/extracellular condition ongoing to malignant transformation (50% of the analyzed patient’s samples developed cancer disease) characterized in Fig. 7 by a down level of the antioxidant defence and by an up level of glycation products in their secretome with respect to the healthy subjects.

In the field of preventive medicine, this study suggests the exciting perspective that would allow to individuate healthy subjects with high cancer risk and to submit them to a personalized monitoring program supported by specific prescriptions or lifestyle guidelines.

Further studies are needed to understand how the pathway identified here can be used to design personalized medicine intervention that may limit, hamper or possibly revert cancer progression.

## Methods

All chemicals, unless mentioned otherwise, were of analytical grade and were used as received. The complete list of chemicals and reagents used in the study is reported in Supporting Table S2.

### Experimental model and subjects details

Blood samples have been collected prospectively from patients at the clinics of the Department of Clinical and Experimental Medicine and the Department of Medical and Surgical Sciences, University 'Magna Græcia' of Catanzaro, from July 2013 up to the present. The study named CHARACTEX (CHARActerization of Circulating Tumor cells and EXpansion) was approved by the local institutional reviewer board and conducted according to the recommendations of the Declaration of Helsinki and its amendments. The study approval number is 2013.34. Enrolled subjects provided written informed consent and were given a patient information sheet detailing the following aspects of the study. Peripheral blood samples (total volume of 5 ml) were drawn from both controls (volunteer healthy subjects) and untreated patients with a primary diagnosis of cancer, placed into tubes containing EDTA as an anticoagulant. All subjects enrolled in this study, had a ‘normal’ glucose level of 70–85 mg/dl and family history without cases of diabetes and/or neurodegenerative diseases. Subsequently, the samples were processed following the procedure described previously by Malara et al.^20^ Patient age, sex, diagnosis and clinical status are described in Data files S1 and S2.

### Liquid biopsy primary culture

To develop the culture, cells of interest were isolated by a working range, as previously reported by Malara et al.^11,12,20^. After washing in phosphate-buffered saline (PBS), the cells were recovered in a medium promoting in vitro expansion for 14 days (short-term cultivation). The medium composition was reported in Malara et al.^11,12,20^

In brief, peripheral blood sample was collected and submitted to a gradient procedure. This phase is useful to reduce the haematological cells contamination. The phase taken during this procedure was enriched for non-haematological cells, as previously reported in cancer cases.^11,12,20^ The isolated solution of cells during the gradient procedure is washed and seeded in a culture plate with the addition of a specific culture medium.^11,12,20^ The cultures were performed for 14 days of cultivation. This timing was assessed to permit the transformed cells to become visible through their self-altered proliferation feature. The same protocol applied in healthy subjects and subjects with inflammatory disease highlighted the presence of non-haematological cells, prevalently endothelial cells able to survive in vitro and with a limited expansion power supported by a lower S-phase.

### Secretome collection and characterization

The cell cultures were monitored at 48-h intervals and each time, 10% of total volume of the culture medium was collected and replaced with fresh medium. The 10% of collected medium was placed in a cuvette and stored at 4 °C. After a 2-week incubation, the culture was harvested and the medium was separated from the cellular elements by centrifugation at 1870 rpm for 15 min. The supernatant was added to the previous collected medium, filtered and stored at −80 °C. The pellet formed was collected and used for successive characterizations.

### Cytometry

Cultivated cells were analyzed with a panel of antibodies designed to identify different populations of naive haematological and non-haematological cells (the list of antibodies used in panel and control tubes is described in Supporting Table S2). The panel included markers non-specific to cancer cells, which, however, provided relevant information in the multipanel context. Cells were fixed and permeabilized using the BD IntraSure kit. All antibodies were titrated to obtain optimal dilution for the present experimental setting. Instrument performances and data reproducibility were checked using Cytometer Setup & Tracking Module (BD) and further validated through acquisition of Rainbow Beads (BD). Compensations were calculated using CompBeads (BD) and single-stained fluorescent cells for DNA dyes (Syto16 and 7ADD). Flow cytometry was performed with a FACS ARIA III (Becton Dickinson) analyzed with FACSDiva v. 6.1.3, and FACSuite v1.05 (BD) software. Cell cycle phases distribution was performed using the Becton Dickinson kit: CycleTEST plus DNA reagent Kit, data acquisition using FACS Canto II (Becton Dickinson) and the analysis was performed with ModFit LT software (http://modfit-lt.software.informer.com/4.0/)

### Immunofluorescence

For cellular immunofluorescent staining, cells on slides were fixed with 4% paraformaldehyde for 15 min at 22 °C. The slides were washed three times with phosphate-buffered saline (PBS) and then blocked with PBS containing 0.2% Triton X-100 and 10% bovine serum albumin for 1 h. Subsequently, the cells were then incubated with primary antibody (1:1000) for 16 h at 4 °C and washed three times with PBS. Next, the cells were incubated with Alexa Fluor 633—or Alexa 488-conjugated secondary antibody for 3 h at 22 °C and viewed using a laser confocal microscope (Nikon TI-E). The TI-E microscope software was used to morphometrically analyze the fluorescence intensity.

### Protein/DNA/RNA assessment

The secretome aliquots were used to assess the protein/DNA/RNA content. Measurements were performed at 280/260-nm wavelength using NanoDrop™ (Thermo Scientific) which allows micro-volume (1 µl) sample measurements and at the same time by a spectrophotometer (Gene Quant *pro*, GE Healthcare. Quartz cell 5-mm pathlength). Each time, three assays were performed on the same sample set for comparison.

### Cytokines and growth factors array

Cytokine and growth factor levels were evaluated simultaneously using the 'Cytokine & Growth Factors Array (CTK)' kit, the Evidence Investigator biochip analyzer (Randox Labs, UK) and by the Proteome Profiler kit (R&D system).

### Isoelectric focusing and immunoblotting

Total protein content of the extracts was determined using the Bradford Protein Assay (Bio-Rad) with human serum albumin (Sigma Aldrich) as standards, according to the manufacturer’s instructions. Fifty micrograms of each proteins extract were diluted with Laemmli buffer and incubated at 100 °C for 5 min. Subsequently, all samples were loaded onto a 12% SDS-polyacrylamide gel and electrophoresed at 50 V. After electrophoretic separation, SDS gel was electrotransferred to a nitrocellulose membrane by Trans-Blot Turbo system (Bio-Rad) protein transfer system. Membranes were incubated using the primary antibody direct against methylglyoxal-modified proteins, lipids and nucleic acid. The detection of primary antibody was done with anti-mouse horseradish peroxidase-conjugated secondary antibodies (Cell Signaling) for mouse primary antibody. Blots were developed using the SuperSignal West Femto ECL substrate (Pierce, EuroClone). Fully automated densitometric software Alliance 2.7 1D (UVITEC, Eppendorf, Milan, Italy) was used to determine the percent distribution of blotted proteins and the image acquisition. Proteins extracts were mixed and resuspended into isoelectrofocusing (IEF) sample buffer containing 8 M urea, 4% CHAPS, 0.1 M DTT and 0.8% IPG buffer, pH 3–10 NL. IEF was performed using a nonlinear precast IPG strip (pH 3–10 NL; 24-cm long, GE Healthcare). The first dimension separation was carried out on an IPGphor unit (GE Healthcare), which to a total of 70 kVh was reached.^25^ After IEF separation, the IPG strip was stained with silver-staining procedure.

### Mass spectrometry analyses

A Thermo Scientific Q-Exactive^TM^ (Rodano, MI, Italy) mass spectrometer was operated using electrospray with both negative and positive polarities at 140000 resolving power and ACG target = 3e6, by SIM (single ion monitoring) analysis. Source conditions were spray voltage 3.4 kV, sheath gas: 30 arbitrary units, probe heater temperature: 280 °C; capillary temperature: 350 °C; S-lens RF level: 50.

### SeOECT chip fabrication

Surface-enhanced organic electrochemical transistors (SeOECT) were fabricated using micro and nano fabrication techniques, as reported in refs.^17,26,27^

### SeOECT chip operation

Samples containing cell culture liquids were gently positioned upon the active surface of the biosensor device in the form of drops of volume *V* < 10 μl. The electrical response of biosensors has been measured using a two-channel source/measure precision unit (Agilent B2902A), controlled by a homemade software. Biosensor measurements were acquired by measuring the drain current Ids versus time under a constant drain voltage Vds = −0.1 V, while varying the voltage at the gate Vgs between 0 V and a positive value that was gradually increased from 0 V to 1 V with a step of 0.2 V, with a time interval of 120 s. Biosensor current response is expressed as current modulation Δ*I*/Io = (I − Io)/Io, where *I* is the drain current value measured for Vgs > 0 V and Io is the Ids value at Vgs = 0 V.

### Statistical analysis

All data are reported as means ± standard deviation with the exception of cytometric data that are reported as means ± standard error. A three-ways analysis of variance (ANOVA) was used to explore the relationship among marker expressions, clinical parameters and cancer characteristics. The significance level was set at *p* < 0.05. Repeated measures analysis of variance (ANOVA) was performed to assess the distributional levels of the cytokines in control and patients’ groups (*p* < 0.05). Comparison between patients and the control group was performed using Mann–Whitney and Kolmogorov–Smirnov tests with a valid statistical significance of *p* < 0.05. In order to rank the correlation and direction of linear relationships between pairs of continuous variables such as cell characteristics and secretome content across patients and controls, we used the Pearson correlation coefficient. Sub-groups were compared using the T test (for continuous variable) and chi-square test or Fisher test (for categorical variables). Follow-up of the patients was defined as the number of months from diagnosis until the first occurrence of loco regional or distant-relapsing lesion or death if any of these occurred. Survival analysis has been conducted using the Kaplan–Meier method, log-rank test. All statistical analyses were performed using MedCalc for Windows, version 18 (MedCalc Software, MariaKerke, Belgium). The heatmap of cytokine levels in MC and TM was displayed in MathLab v.R2016 b. Each cytokine level, assessed with a proteomic array kit (R&D system), represents the average of the two values of pixel intensity quantified on spots of the cytokine-array membranes with ImageJ software.

Cytometry data were analyzed using a multivariate mathematical approach in order to identify several cellular patterns across the different patients. For this purpose, 5000 cells representing grade I, grade II, grade III, grade IV patients and healthy individuals (summing up to 25,000 cells) were extracted from FACS data. For each cell, the scattering intensities of ten fluorophores were recorded. A principal component analysis (PCA) was applied to the whole fluorimetric data set.

### Analysis of variance of biochip data

Modulation and time constant variables acquired for the entire population of control (C), patients (P) and intermediate (I) samples (data set) were subjected to Multifactorial ANalysis Of VAriance (ANOVA), in order to individuate the experimental variables significantly correlated to the sample label. ANOVA was performed using the Statgraphics Centurion XV statistical software (Statpoint Technologies Inc., Warrenton, VA, USA). In the ANOVA analysis, the modulation and time constant outputs from the five sensors (pooled in this stage of data management) were considered. Intermediate samples were intentionally excluded from the model development to improve its predictive power. The classification factors for ANOVA were the applied gate potential and sample typology. Bonferroni post hoc test on time constant and modulation outputs was carried out considering as significant the factors with *p* < 0.01. Two-way ANOVA interaction plots of time constant outputs and modulation outputs were realized according to the significance found in the test, reporting the mean value and the Bonferroni confidence intervals (*p* = 0.01).

### Principal component analysis of biochip data

On the basis of ANOVA results, PCA was carried out both for modulation and time constant outputs, including independent outputs from the different five sensors and excluding from the data set the measurements performed at Vgate values non-significantly associated to the 'label' of the samples, in order to avoid the introduction of 'noise' in the data-modelling procedure. The entire statistical analysis is described in detail in Supporting Information 2 and 3.

## Electronic supplementary material

Supporting Information

Data file S1

Data file S2

## Data Availability

The authors declare that all data supporting the findings of this study are available within the paper and its Supplementary Information files and the raw data are available from the corresponding author upon reasonable request.

## Electronic supplementary material

**Supplementary Information** accompanies the paper on the *npj Precision Oncology* website (10.1038/s41698-018-0069-7).
