# Supplementary material for: Superhydrophobic lab-on-chip measures secretome protonation state and provides a personalized risk assessment of sporadic tumour
Source: NPJ Precis Oncol. 2018 Nov 19;2:26. doi: 10.1038/s41698-018-0069-7 (PMC6242957; doi:10.1038/s41698-018-0069-7)
Supplement: Supplementary file 1 — Supporting Information [file 41698_2018_69_MOESM1_ESM.pdf]

Supporting Information 1: *Cellular elements included in blood-derived cultures (BDCs) across control and patients.*

Evaluation of cellular elements included in blood-derived cultures (BDCs)

Prospective project CHARActerization of Circulating Tumor cells and EXpansion (CHARACTEX) enrols oncologic patients at University Magna Graecia form 2013. 275 patients diagnosed with all grade and stage of cancer were enrolled to monitor circulating tumour cells (CTCs) and /or to assess treatment planning and resistance, and 57 volunteer healthy subjects for early cancer diagnosis (update 2017). Sixty subjects without glucose dysmetabolism were selected from CHARACTEX-cohort (**Data file S1**). Twenty-four healthy subjects (12 female, 12 male) constituted control group (mean age 44ys), and forty patients (29 female and 11 male) with a diagnosis of cancer (mean age 58ys). Clinical data are detailed in Data file S1 and S2 respectively. According to our original protocol[13], we isolated CTCs by working-cell-phase previously identified for colon, lung, breast, pancreas and glioblastoma[8,13,14]. The cells isolated from working-phases were seeded in a specific culture medium and expanded for 14ds. First screening on expanded cells was non-haematological ($CD45^{neg}$) and haematological cells ($CD45^{pos}$) phenotype evaluation (**Supporting Figure S1b-c**). In control group the media of percentage (media ± Standard deviation, SD) of $CD45^{pos}$ was of 94±4, and $CD45^{neg}$ was of 6±4. In the patient's group was of 37±12 $CD45^{pos}$ and 63±12 $CD45^{neg}$. Comparative analysis between the two clinical groups showed a highly significant difference of p=0,004 for prevalent $CD45^{neg}$ in the patients 'group[15]. Moreover, within the patients group $CD45^{neg}$ cells increased in patients with high histological grade (p=0,02) (**Supporting Figure S1b**).

Analysis of phenotype and proliferation rate in BDCs across patients.

Cytometric analysis on population of $CD45^{neg}$ found in the patients' BDC was performed (**Supporting Figure S1d-e**). The marker panel adopted is not exclusive to cancer cells. Nevertheless, it is a valid tool in the context of the multipanel approach. Each marker was analysed in order to estimate the mean of percentage of cells positive (mean ± Standard error of

the mean) and the mean fluorescence intensity (MFI) quantified after a titration curve optimized for each antibody used in the panel. PCA-maps displaying 5000 cells in patients and control BDCs (**Supporting Figure S1d**). Events corresponding to circulating cancer cells were grouped in P2-8 clusters and in P1 were grouped events corresponding to circulating non haematological cells. Each cluster is coloured according to their normalized markers expression on PCA-maps. The endothelial phenotype $CD45^{neg}CD146^{pos}$ was presented in a mean of 4,39± 0,6834 cells particularly in NSCLC and glioblastoma (r=0, 36). $CD45^{neg}$Pan-CK^{pos} and $CD45^{neg}CD326^{pos}$ phenotype was recognized in a mean of 23±2,6 and of 4±0,5 cells prevalently in breast, colon, lung and thyroid tumours (r=-0,5). $CD45^{neg}Vimentin^{pos}$ and $CD45^{neg}Fibronectin^{pos}$ markers was found in a mean of 10±1,4 and 10±1 cells with a prevalence of vimentin in melanoma and glioblastoma (r=0,5) and fibronectin in breast cancer (r=0,4). Cancer stem like phenotype, $CD45^{neg}CD44^{pos}$ in a mean of 2,7±0,4 and $CD45^{neg}CD133^{pos}$ in 2±0,3 cells and their expression increased with the grade (r=0,4). Epithelial mesenchymal transition phenotype $CD45^{neg}$Pan-$Ck^{pos}Fibronectin^{pos}$ was found in 5,6±1,1 cells increasing with grade and stage (r=0,5 and r=0,6). The analysis of proliferation rate of the expanded cells were performed with the cytometric evaluation of the cell cycle phase distribution focusing on the S-phase as indicator of the percentage of cultivated circulating cells ongoing to cell-division. In BDC of cancer patients showed an S-phase of 52,7583 to 62,8767(%) at 95% of confidence interval a standard deviation of 15,8 correlated with the grade and stage of disease (r=0,5 and 0,6) (**Supporting Figure S1f-g**).

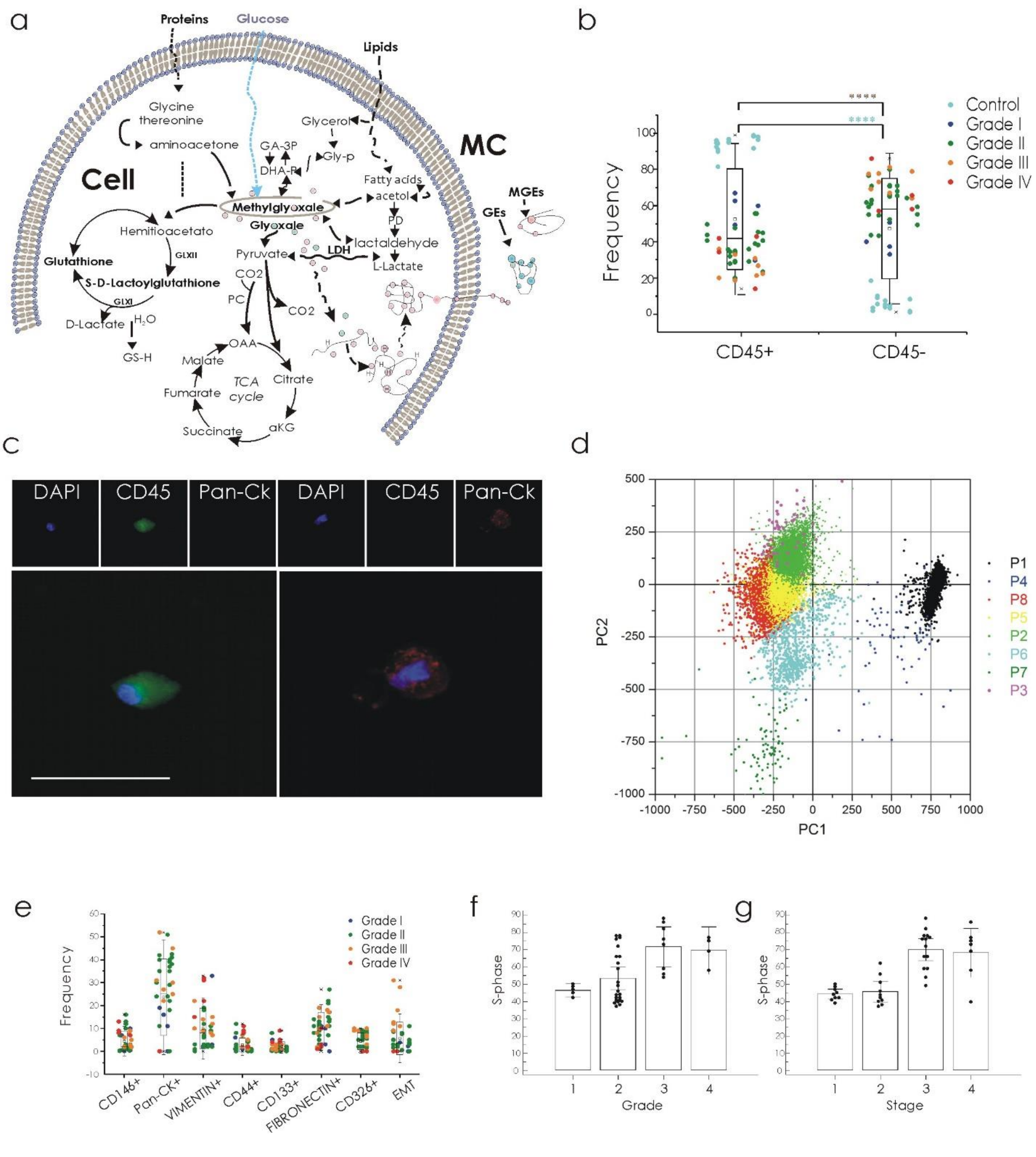

Supporting Figure S1. ***Metabolic, phenotypic and characterization of cellular elements included in bloodderived cultures***. *A) Pathway through which 2-Oxos, methylglyoxal and glyoxal, formed during metabolisms leading to the formation of glycation-end-products. B) Prevalence of CD45neg cells (p=0,004) in BDCs of cancer patients C) Haematological (CD45pos) and non-haematological (CD45negPanCKpos) cells cultivated in vitro. D) PCA-maps displaying events corresponding to circulating cancer cells grouped in P2-8 clusters and circulating non haematological cells in P1. Each cluster is coloured*

according to their normalized markers expression on PCA-maps E)Box and Whisker Plot detailing the frequency of CTCs grouped in grade. F-G) Distribution of cell cycle S-phase percentage in cancer BDC. Scale bars 100 mm

Supporting Information 2 *Clustering analysis of a second cohort of 9 samples*

A cohort of 9 subjects signed by ** in Data file S1 were analysed by SeOCET. This cohort were composed by 5 subjects affected by non-cancerous inflammatory disease and 4 healthy subjects. Data suggested that the cultivated cells isolated from the liquid biopsy performed in subjects with no inflammation displayed a higher Ps rather than the cancer patients and were grouped within the subset of control samples.

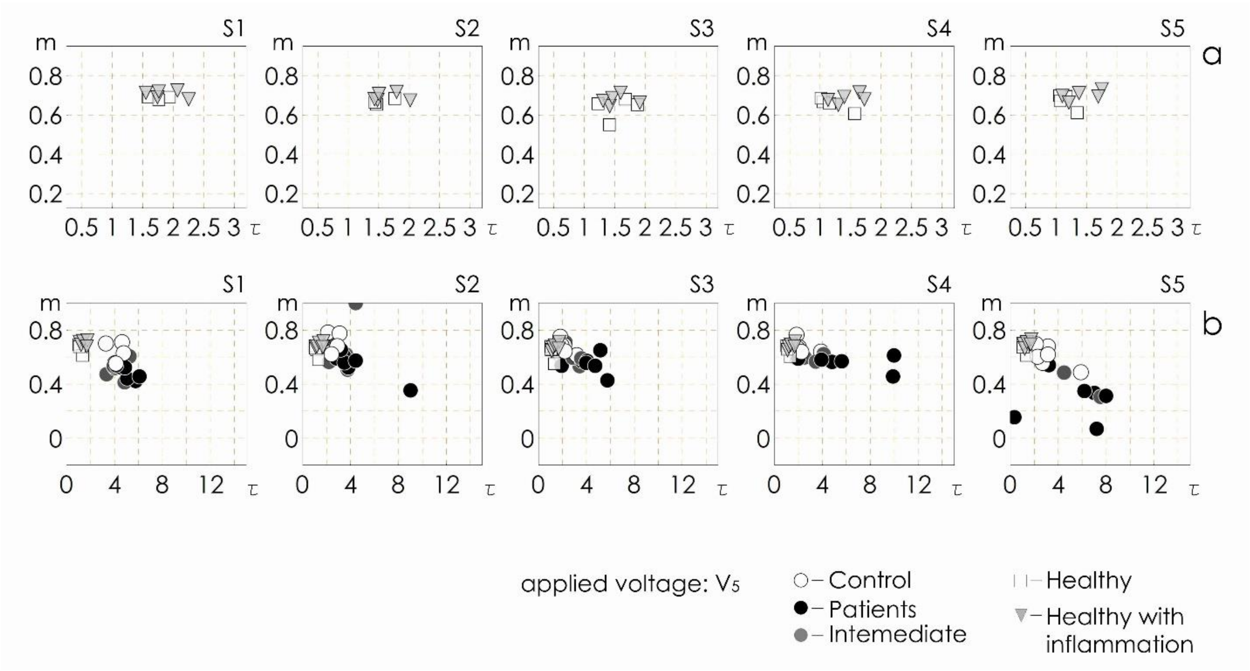

Supporting figure S2A. All sensors and voltage V5

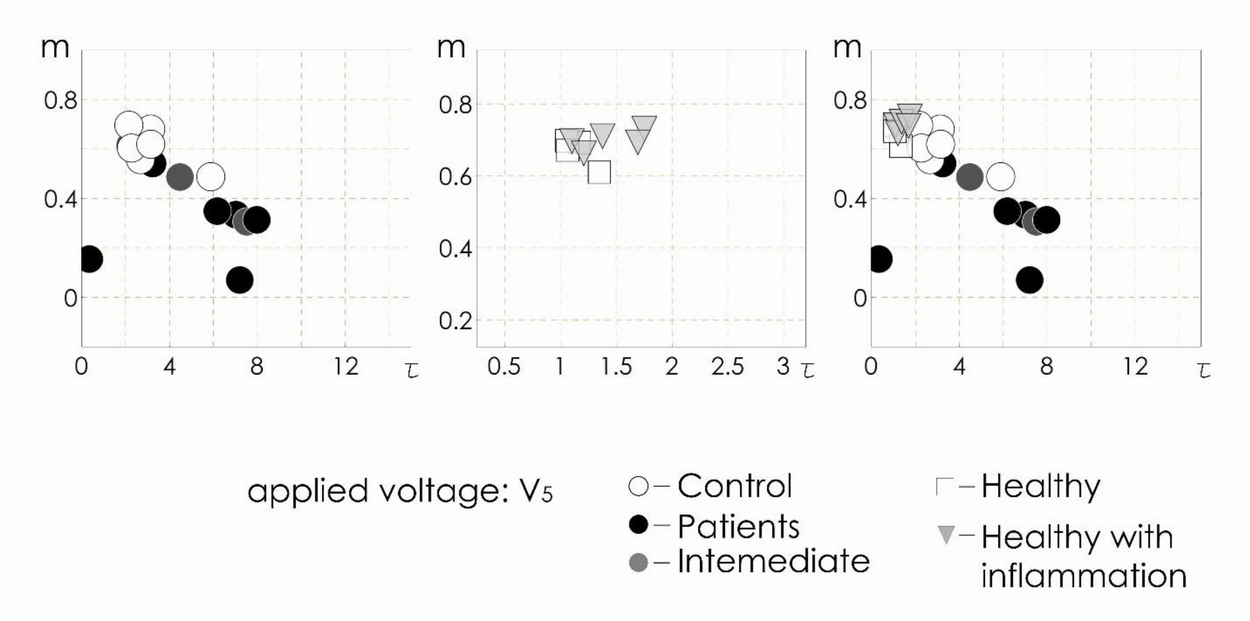

Supporting figure S2B: sensors 5 and voltage V5

Supporting Information 3 *Statistical analysis of SeOECT data*

Principal Components Analysis of SeOECT data. On the basis of ANOVA results, PCA was carried out both for modulation and time constant outputs, including independents outputs from the different five sensors and excluding from the dataset the measurements performed at Vgate values non significantly associated to the “label” of the samples, in order to avoid the introduction of “noise” in the data modelling procedure. Also in this case only “C” (control) and “P” (patients) samples were included in the analysis. PCA performed on modulation outputs acquired at V3, V4 and V5 Vgate values gave good result in terms of PC extraction, as the eigenvalues resulted >1 for the first 3 components, with a cumulative explained variance of 95.7 % (**Supporting Table S3.1, S3.2**). PCA performed on tau outputs acquired at V4 and V5 Vgate values also gave good result in terms of PC extraction, as the eigenvalues resulted >1 for the first 2 components, with a cumulative explained variance of 92.4 % (Table S4). The weights of the single variables on the extracted components were used in order to select the “best six” experimental outputs, in terms of discrimination capability among C and P values. A matrix scatterplot of the modulation outputs extracted from PC1 (**Supporting Figure S3a**) evidences not only a good clustering of C and P samples but also a good placement of the intermediate samples, so indicating the high prediction power of the model. The matrix scatterplot of the modulation outputs extracted from PC2 (**Supporting Figure S3b**) also evidences a very good sample clustering. Analogously, the matrix scatterplot of the time constant outputs extracted from PC1 (**Supporting Figure S3c**) and PC2 (**Supporting Figure S3d**) evidences the high predictive power of time constant values.

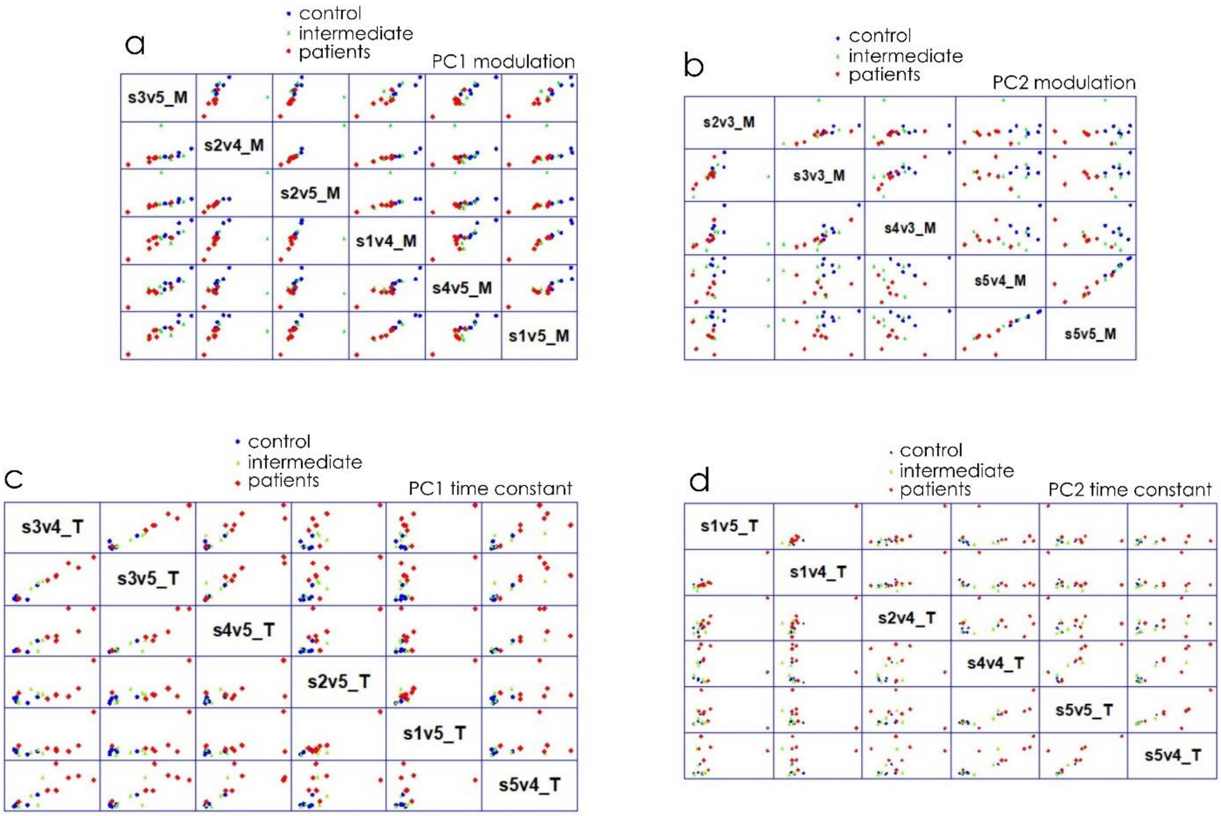

Supporting Figure S3. ***PCA analysis*** *allows to reduce the dimensionality of a data set and extract the variables that more contribute to its variation. Here, we show the best six combinations of sensor number S and voltage V resulting from sorting the first PC1 and second PC2 principal components extracted from the modulation (a-b) and time constant (c-d) output of the device.*

i^{th} component	Eigenvalue	Percent of variance	Cumulative Percentage	variable	PC1	PC2	PC3
1	11.4402	76.268	76.268	*s1v3*	0.190022	-0.0515569	-0.689042
2	1.78469	11.898	88.166	*s1v4*	0.285689	-0.0162998	-0.18482
3	1.13313	7.554	95.720	*s1v5*	0.282102	0.0615145	-0.210798
4	0.294974	1.966	97.686	*s2v3*	0.260835	-0.212158	-0.285159
5	0.162759	1.085	98.771	*s2v4*	0.286176	-0.119242	-0.0451314
6	0.113434	0.756	99.528	*s2v5*	0.286119	-0.00370142	-0.149452
7	0.0303883	0.203	99.730	*s3v3*	0.246771	-0.355175	0.126657
8	0.0229278	0.153	99.883	*s3v4*	0.277697	-0.157418	0.231658
9	0.008542	0.057	99.940	*s3v5*	0.289525	0.0285232	0.0313399
10	0.00559911	0.037	99.977	*s4v3*	0.242603	-0.301909	0.327332
11	0.00319476	0.021	99.999	*s4v4*	0.26761	-0.108955	0.33615
12	0.000203604	0.001	100.000	*s4v5*	0.282603	0.0636058	0.142628
13	0.0	0.000	100.000	*s5v3*	0.233629	0.407585	0.0863358
14	0.0	0.000	100.000	*s5v4*	0.229227	0.431557	0.146506
15	0.0	0.000	100.000	*s5v5*	0.177754	0.568219	0.0290691

Supporting Table S3.1 ***PCA modulation variables***. *Principal components analysis performed on modulation outputs acquired at V3, V4 and V5 values of Vgate. We show, for the first 15 components, eigenvalue number, percentage and cumulative percentage of variation, and associated first, second and third principal components. We observe that the first 11 components retain the 99.999% of the information content of the output signal.*

i^{th} component	Eigenvalue	Percent of variance	Cumulative Percentage	variable	PC1	PC2
1	6.07762	60.776	60.776	*s1v4*	0.30065	-0.366565
2	3.1625	31.625	92.401	*s1v5*	0.328011	-0.31955
3	0.528367	5.284	97.685	*s2v4*	0.314965	-0.2595
4	0.162881	1.629	99.314	*s2v5*	0.356354	-0.256252
5	0.027911	0.279	99.593	*s3v4*	0.385435	0.12205
6	0.0182766	0.183	99.776	*s3v5*	0.379521	0.155319
7	0.0132279	0.132	99.908	*s4v4*	0.219424	0.465654
8	0.00674187	0.067	99.975	*s4v5*	0.357639	0.0926309
9	0.00212805	0.021	99.997	*s5v4*	0.316689	0.330719
10	0.000341372	0.003	100.000	*s5v5*	0.0829861	0.506659

Supporting Table S3.2 ***PCA time constant variables****. Principal components analysis performed on time constant outputs acquired at V4 and V5 values of Vgate. We show, for the first 10 components, eigenvalue number, percentage and cumulative percentage of variation, and associated first PC1 and second PC2 principal components. We observe that the first 9 components retain the 99.997% of the information content of the output signal.*

Supporting Information 4. *The clustering algorithm*

We partitioned elements into groups using a density based clustering algorithm[18]. The algorithm classifies elements into categories on the basis of their similarity. Cluster centers are determined as those points in the set with higher density than their neighbors and by a relatively large distance from points with higher densities. To do so, per each point o in the set:

(i) we determine its density $\rho(o)$ as the number of points that are closer than a cut off distance δ_{co} to o;

(ii) find the subset $s \epsilon S$ of points in the dataset with densities $\rho(s) > \rho(o)$;

(iii) find the point $a \epsilon S$ with minimum distance to o, this distance is $\delta_{min}(o)$: the minimum distance of o from points with higher densities than o.

After operations from (i) to (iii), we derive a diagram where the density ρ is reported against $\delta_{min}(o)$ per each element in the data set. Points in the set with higher density than their neighbors and by a relatively large distance from points with higher densities emerge as singularities in the diagram, an example of which is reported in Supporting figure S4.1. These points are the cluster centers. Each point in the set is attributed to different clusters on the basis a minimum distance criterion: a point b is attributed to a cluster G_i if the minimum distance of b to G_i is the smaller among all the minimum distances calculated with the remaining clusters. Thus clusters are constructed per accumulation. The cluster centers represent the seeds of the clusters.

Here, we found that the totality of tumor and non-tumor samples is partitioned in the modulation/time plane into two separate groups – with all tumor samples gathered in the same cluster (say A), and all non-tumor samples gathered in the other clusters (say B). This is relevant because unsupervised clustering (i) finds, without prior knowledge, that there are two groups with some internal correlation in the data set (reflecting the initial number of sample categories, i.e. tumor and non-tumor) and (ii) associates all elements of a category to the same cluster (revealing that clusters have internal consistency and that clustering reflects physical differences between categories).

Suppose now to have A and B (*actually*, we have A and B – they are the distributions of measured tumor and non tumor samples as in Figures 3f and 5 of the main text). Then, we have an additional measure of the unknown (undetermined) sample c. Suppose that c is a tumor sample. If c falls within the convex closure of A, then the algorithm would assign p to the correct cluster A with 100% confidence. If p falls outside the convex closure of A, we can assign c to A with probability p and c to B with probability $e = 1 - p$. The closer c to the border of B, the greater e. We can calculate p and e on a statistical basis.

We assume c is drawn by a Gaussian distribution with standard deviation $\sigma(A)$ and mean $\mu = \bar{x}(A) \pm z\ \sigma(A)/\sqrt{n}$ (n is the size of A, μ is the mean of the population, $\bar{x}$ is the mean of sample A, $\sigma(A)/\sqrt{n}$ is the standard error of the mean z is the score associated to specific confidence intervals).

We generate a large number N of tentative $c's$ ($N > 1000$ Supporting Figure S4.2a). Then, we examine whether c falls in the first ($c \rightarrow A$) or in the second ($c \rightarrow B$) group. p is determined as the number of $c \rightarrow A$ events to N. Supporting Figure S4.2b reports p as a function of the size of sample A for different values of the confidence interval z. The method assigns the unknown sample to the correct cluster with 100% reliability ($p = 1$) and 0% uncertainty ($e = 0$) for any initial sample with size $n > 2$ and fixed confidence interval $CI < 98\%$ ($z = 2.33$). Fixing the confidence interval to $CI = 99.99\%$ ($z = 3.29$), the size of A necessary for reaching 100% increases to $n \sim 15$. Thus, for any sufficiently large initial tumor set, the method would diagnosis unknown, potentially tumor samples deterministically, i.e. with $e = 0$. The analysis, here reported for the couple of variable $S_3\tau - S_5 m$, can be performed for any combination of modulation, time constant, and sensor number that maximize the system response, resulting from PCA post-processing of data.

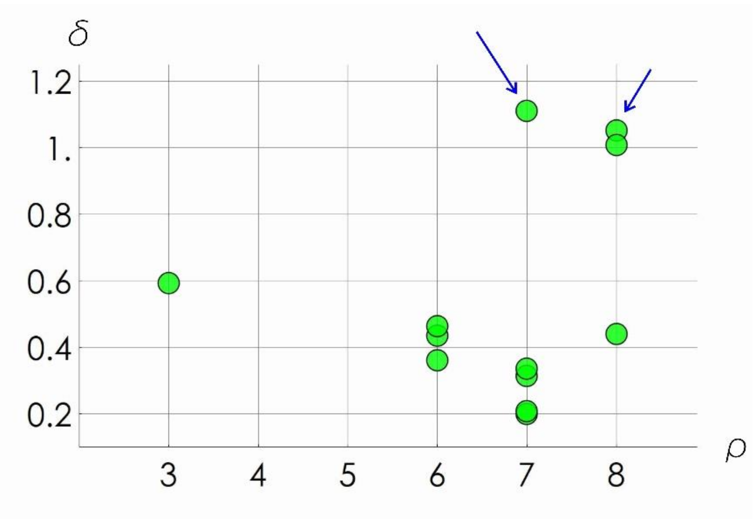

Supporting Figure S4.1

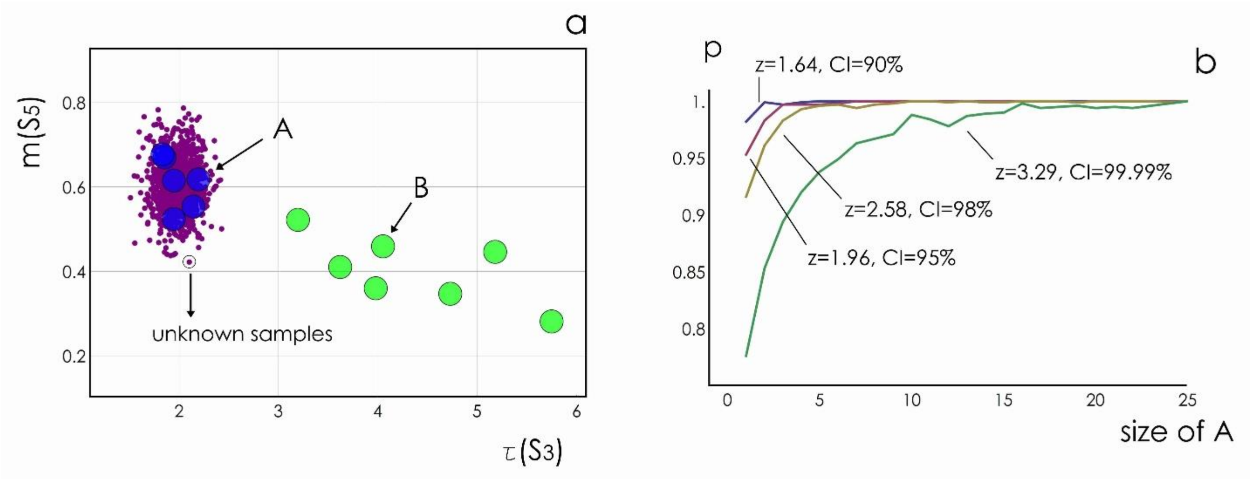

Supporting Figure S4.2

Supporting Information 5 *Clustering analysis of a third cohort of 8 samples*

In addition to data presented in the main text, we here show results relative a cohort of 8 samples, independently measured by the OECT device. The samples are constituted by 2 control subjects, 6 patient subjects. Scatter plots of modulation vs time constant of the output of the sensor are reported in the Supporting Figure S5, measured at the sensor number S2 and voltage V4 (a), sensor number S3 and voltage V5 (b), sensor number S5 and voltage V4 (c). Clustering of data using non Euclidean metrics as explained in the main text, enables data classification of

the sole patient (tumor) and control (healthy) subjects with 100% performance for case (a) and (b), and 87% performance for case (c).

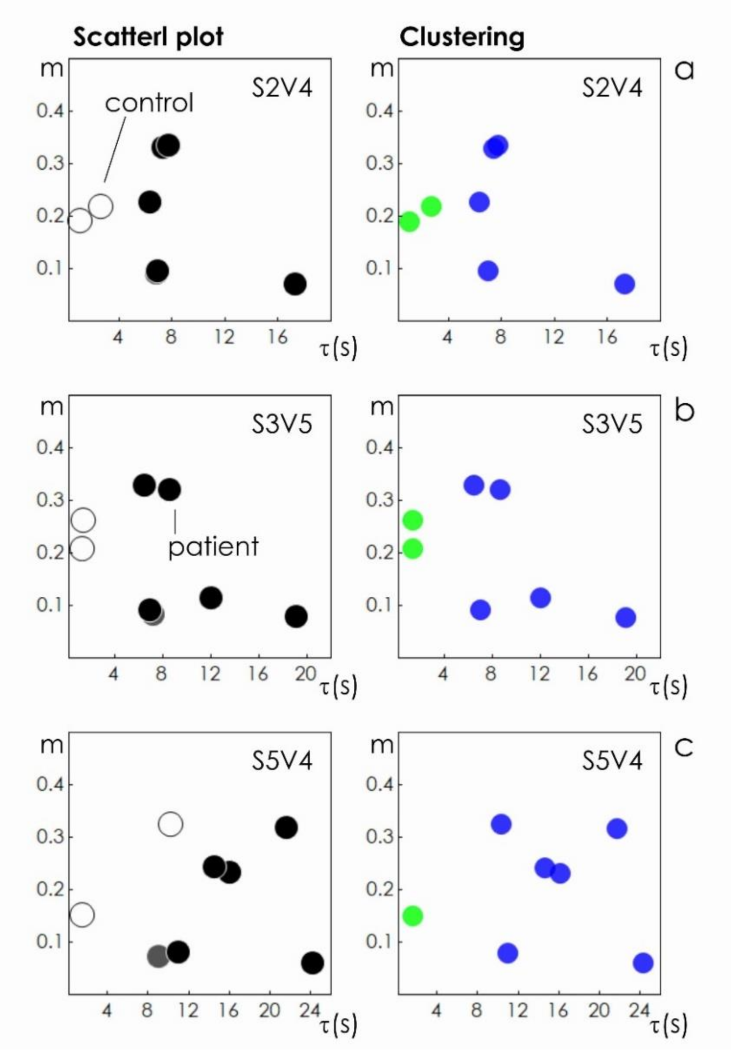

Supporting Figure S5

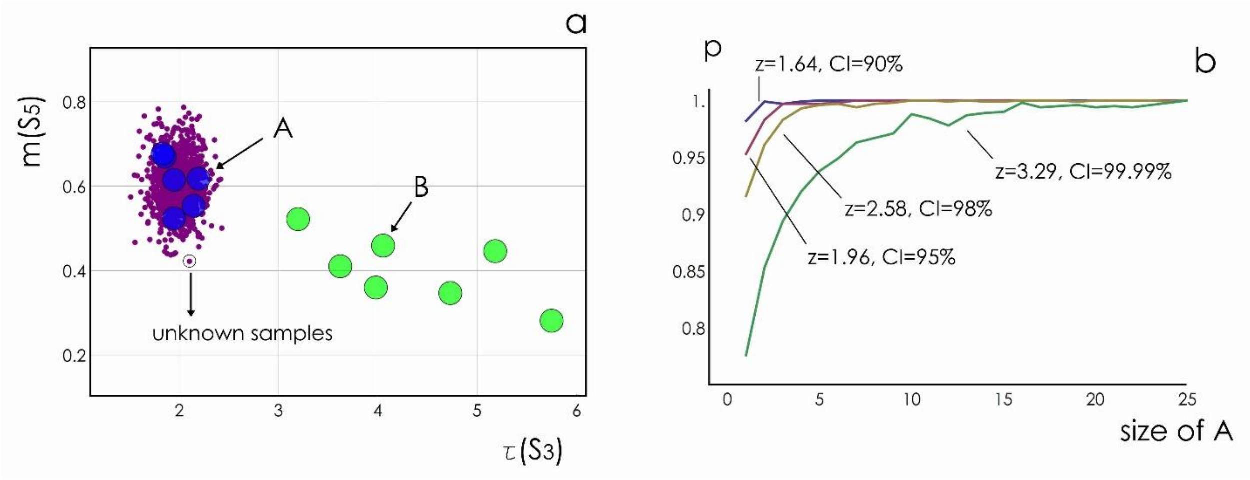

Supporting Figure S5.2

Supporting Tables

Analyte	Neutral mass (Da)
Lysine	147.1
MetSO	166.1
CEL	219.2
FL	291.0
Arginine	175.2
GOLD	327.1
MG-H1	229.2
MOLD	341.2
G-H1	215.0
Tyrosine	182.1
Dityrosine	361.2
Methionine	150.0
3-Nitrotyrosine	227.1

Supporting Table S1 ***Detection of protein biomarkers through Mass-spectrometry.*** *MG-derived AGEs (MGH1,CEL, MOLD and argpyrimidine) and Glyoxal-derived AGEs (G-H1, CML and GOLD) analysed by MS to extimate the different protein glycation behavieour related to disease presence. Abbreviations. Methionine sulphoxide (MetSO); Fructosyl-lisine residues (FL), N-(5-hydro-5-methyl-4-imidazolon-2yl)-ornithine (MG-H1); N-(1-Carboxyethyl)lysine (CEL); Nδ-(5-hydro-4-imidazolon-2-yl)ornithine(G-H1), N-(1-Carboxymethyl)lysine (CML) and (H8)-GOLD; methylglyoxal-derived lysine dimer (GOLD)*

REAGENT or RESOURCE	SOURCE	IDENTIFIER
**Antibodies**		
CD133	Clone 293C3 MACS	Cat# 130-104-322
CD326	Clone EBA-1 Becton Dickinson	Cat# 563180
CD44	Clone G44-26 Becton Dickinson B	Cat# 562991
CD146	Clone P1H12 Becton Dickinson	Cat# 564327
CD45	Clone 2D1 Becton Dickinson	Cat# 564327
FIBRONECTIN	Clone10/Fibronectin Becton Dickinson	Cat# 563098
VIMENTIN	Clone RV202 Becton Dickinson	Cat# 562338
PANCK	Clone C-11 ABCAM	Cat# Ab106166
7-AAD	Life Technology	Cat# A1310
SYTO 16	Life Technology	Cat# S7578
Isotype CD133	MACS	Cat#130-092-212
Isotype PANCK	Clone 1F8 ABCAM	Cat#ab91358
CD34	Becton Dickinson	Cat# 340441
CD49F	Becton Dickinson	Cat# 562952
CD184	Becton Dickinson	Cat# 557907
CD24	Becton Dickinson	Cat# 550927
Anti-Methylgliox-adducts	BIOLABS	Cat# STA-011
Anti-mouse IgG, HRP-linked Antibody	Cell Signaling	Cat# 7076
Anti-p21	Cell Signaling	Cat# 0005
Anti-rabbit IgG	Cell Signaling	Cat# A21070
Anti-mouse IgG	Thermofisher	Cat# A-21052
**Cell lines**		
CAL 62	DSMZ	ACC-448
SKMEL-24	ATCC	HTB-71
**Critical Commercial Assays (KIT)**		
CycleTEST PLUS DNA Reagent Kit	BD Becton Dickinson	Cat# 340242
Profiler Cytokine Array Panel A	RD Systems	Cat# ARY005
IntraSure Kit	BD Becton Dickinson	Cat# 641778
Bradford Protein-Assay Kit II	BIORAD	Cat# 5000002
Cytokine & Growth Factors Array (CTK)	Randox Labs, UK	Cat# EV 3513

Supporting Table S2. ***List of chemicals and reagents***
